# Enhanced production of d-pantothenic acid in *Corynebacterium glutamicum* using an efficient CRISPR–Cpf1 genome editing method

**DOI:** 10.1186/s12934-023-02017-1

**Published:** 2023-01-06

**Authors:** Rui Su, Ting Wang, Taidong Bo, Ningyun Cai, Meng Yuan, Chen Wu, Hao Jiang, Huadong Peng, Ning Chen, Yanjun Li

**Affiliations:** 1 College of Biotechnology, Tianjin University of Science and Technology, Tianjin, 300457 China; 2 Key Laboratory of Industrial Fermentation Microbiology, Ministry of Education, Tianjin University of Science and Technology, Tianjin, 300457 China; 3 The Novo Nordisk Foundation Center for Biosustainability, Technical University of Denmark, 2800 Kongens Lyngby, Denmark

**Keywords:** *Corynebacterium glutamicum*, CRISPR-Cpf1, Genome editing, Vitamin, Metabolic engineering, Synthetic biology

## Abstract

**Background:**

*Corynebacterium glutamicum* has industrial track records for producing a variety of valuable products such as amino acids. Although CRISPR-based genome editing technologies have undergone immense developments in recent years, the suicide-plasmid-based approaches are still predominant for *C. glutamicum* genome manipulation. It is crucial to develop a simple and efficient CRISPR genome editing method for *C. glutamicum*.

**Results:**

In this study, we developed a RecombinAtion Prior to Induced Double-strand-break (RAPID) genome editing technology for *C. glutamicum*, as Cpf1 cleavage was found to disrupt RecET-mediated homologous recombination (HR) of the donor template into the genome. The RAPID toolbox enabled highly efficient gene deletion and insertion, and notably, a linear DNA template was sufficient for gene deletion. Due to the simplified procedure and iterative operation ability, this methodology could be widely applied in *C. glutamicum* genetic manipulations. As a proof of concept, a high-yield D-pantothenic acid (vitamin B5)-producing strain was constructed, which, to the best of our knowledge, achieved the highest reported titer of 18.62 g/L from glucose only.

**Conclusions:**

We developed a RecET-assisted CRISPR–Cpf1 genome editing technology for *C. glutamicum* that harnessed CRISPR-induced DSBs as a counterselection. This method is of great importance to *C. glutamicum* genome editing in terms of its practical applications, which also guides the development of CRISPR genome editing tools for other microorganisms.

**Supplementary Information:**

The online version contains supplementary material available at 10.1186/s12934-023-02017-1.

## Background

*Corynebacterium glutamicum* is a nonpathogenic and safe strain and does not produce endotoxins [1]. Wild type *C. glutamicum* ATCC 13,032 has been engineered to screen high-yield industrial strains owing to its excellent protein expression system [2, 3]. *C. glutamicum* has been favoured by researchers and industry because it is an important industrial bacterium being used to produce amino acids, organic acids, vitamins, and biofuels [4–6]. For example, d-pantothenic acid (D-PA, also known as vitamin B5) is the key precursor of coenzyme A and an acyl carrier protein [7] which can be synthesized by microorganisms and plants, but not by humans or other animals. D-PA has been widely used as a feed and food additive, as well as an active ingredient in the cosmetic and pharmaceutical industries [8]. The commercial production of D-PA occurs via chemical synthesis routes, which involve highly toxic raw materials and cumbersome optical resolution besides causing pollution through cyanide-contaminated wastewater [7]. Hence, it is necessary to develop microbial cell factories for D-PA production and it is used as a proof-of-concept in this study.

The traditional and predominant genome editing technologies currently used for *C. glutamicum* are complicated and time consuming, because they depend on two rounds of homologous recombination (HR) and require a non-replicating vector (e.g., pk18*mobsacB*) with a counter-selectable marker to select the target mutants [9]. To address this issue, fast developed clustered regularly interspaced short palindromic repeat (CRISPR) genome editing technologies have successfully been applied in *C. glutamicum* and other species [10–13]. The well-studied endonucleases include Cas9 from *Streptococcus pyogenes* (*Sp*Cas9) [12] and Cpf1 from *Francisella novicida* (*Fn*Cpf1) [14]. *Fn*Cpf1 was selected in the pioneering work of CRISPR genome editing of *C. glutamicum* because *Sp*Cas9 was found to be toxic [15] and the codon-optimized *cas9* gene for *Actinomycetes* was reported suitable for *C. glutamicum* [16]. The CRISPR interference (CRISPRi) technology was established in *C. glutamicum* before CRISPR-based genome editing [17]. Subsequently, several studies have advanced the application of CRISPR/Cas9 technology in *C. glutamicum* [18–24]. Point mutation(s) in the *C. glutamicum* genome can be achieved efficiently using the CRISPR-based RecT-assisted single-stranded DNA recombineering techniques [15, 18]. In which, additional synonymous mutations must be introduced into the protospacer and protospacer adjacent motif (PAM) regions to avoid Cas9/Cpf1 cutting. This limitation of the CRISPR–RecT system may be bypassed by using base editing [22, 25]. In particular, gene deletions and insertions are more frequently performed in engineering *C. glutamicum*. However, the efficiencies of available CRISPR-based genome editing methods for gene deletions/insertions remain relatively low, especially for the large gene insertions, and a plasmid-borne template is still a mandatory requirement [21, 25]. These drawbacks leave room for the development and improvement of the CRISPR-based genome editing methods in *C. glutamicum.* Therefore, to reduce the time and cost taken for strain engineering design-build-test-learn (DBTL) cycle, it is crucial to develop a simple and efficient CRISPR genome editing method for *C. glutamicum*.

In this study, firstly, we developed a one-step RecET-assisted CRISPR–Cpf1 genome editing method for *C. glutamicum*. Owing to the improved HR activity by RecET and strong expression of *Fn*Cpf1, this method allowed for efficient gene deletion and insertion. Then, we proposed a two-step editing method that harnessed the CRISPR-induced double-strand break (DSB) as a counterselection. This RecombinAtion Prior to Induced Double-strand-break (RAPID) methodology enabled gene manipulations with high efficiency. What’s more, to demonstrate the advantages of the RAPID method, the proof-of-concept D-PA producing strain was constructed and achieved the highest titer of 18.62 g/L when using glucose as main carbon source without β-alanine addition. Collectively, this system could pave the way for practical routine manipulations of the *C. glutamicum* genome.

## Results and discussion

### Selection of recombinases

Current CRISPR genome editing technologies rely on HR between the donor DNA and the genome; the addition of recombinases to the toolbox considerably enhances gene editing efficiency. In widely used *E. coli* genome editing systems, the recombinase Redαβγ system from phage λ has often been exploited [26, 27]. The endogenous recombination pathways in *C. glutamicum* are inefficient [16], and the inclusion of recombinases in CRISPR-based editing systems has not been widely reported for *C. glutamicum*. As dsDNA recombination efficiency was found to be low with the help of RecE and RecT from *E. coli* [28], Jiang et al. claimed that Red/ET recombination systems from native or closely related organisms need to be explored in *C. glutamicum* [15]. RecA has been shown to improve λ-Red recombination efficiency in *E. coli* [29, 30]. In this study, we compared the HR efficiencies of different recombinases including Redαβγ, RecET, RecA and RecET-RecA, and used kanamycin as a genetic marker, which enables rapid screening of genome-edited strains. We first constructed two starting strains, entitled *Cg*Del and *Cg*Int, which were used for rapid identification of gene deletion and integration, respectively. *Cg*Del was used for screening colonies with the correct gene deletion, since removing *xylA*_Eco_* from the kan^R^ cassette confers kanamycin resistance (Additional file 1: Figs. S1a, S2a). *Cg*Int was used to investigate the genomic integration of DNA fragments of different lengths (approximately 500, 1000, 2000, 3000, 4000, and 5000 bp). When the 193 bp C-terminal of the kan^R^ sequence was complemented, together with additional sequences of different lengths (Additional file 1: Fig. S1b), the expression of active kan^R^ facilitates the easy identification of cells (Additional file 1: Fig. S2b). In addition, colony PCR was performed for the verification of *xylA*_Eco_* deletion from *Cg*Del, and the results were consistent with those of colony growth on agar plates supplemented with kanamycin (Additional file 1: Fig. S2c), indicating the feasibility of kanamycin selection for genome-edited strains.

**Fig. 1:**
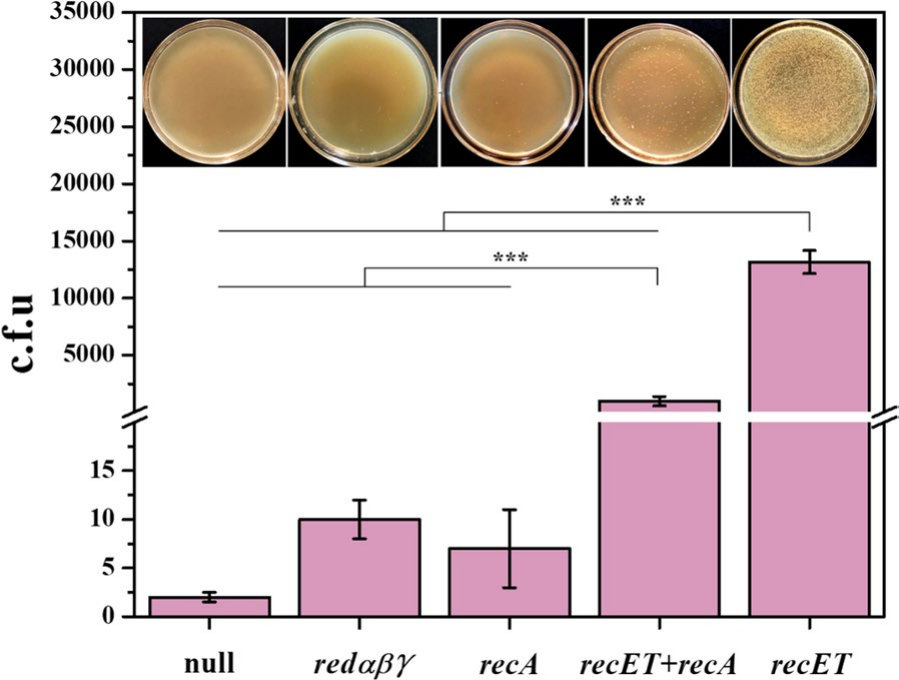
Effects of the expression of different recombinases on HR of DNA fragment into the genome. A 528 bp DNA fragment flanked by approximately 500 bp genomic-homologous upstream and downstream arms was used. The integration efficiency was reflected by the colonies formed on kanamycin agar plates, which were counted or calculated based on dilution. Experiments were performed in triplicates. Values are presented as mean ± SD. ***P < 0.005

Four recombinase-expressing plasmids, pEC-Redαβγ, pEC-RecET, pEC-RecA, and pEC-RecET-RecA were constructed, in which *redαβγ*, *recET* and *recA* were expressed under the control of the weak promoter P_hom_ [31]. The 528 bp fragments fused with the upstream and downstream arms (Additional file 1: Fig. S1b) were electroporated into the competent cells of the model strain *Cg*Int harboring different recombinase-expressing plasmids, and colonies grown on agar plates supplemented with kanamycin were counted or calculated based on dilution (Fig. 1). Only a few transformants possessing kanamycin resistance appeared when Redαβγ and RecA were used. In contrast, there were approximately 1.3 × 10^4^ colonies per plate in the RecET-assisted group, which, in principle, was sufficient for CRISPR-based genome editing. But the combination of RecA and RecET drastically reduced the HR efficiency. Therefore, the expression of RecET only was selected for further experiments.

### One-step RecET-assisted CRISPR–Cpf1 gene deletion and insertion

Although the *F. novicida* (Fn) CRISPR–Cpf1 system has been adapted for gene deletion in *C. glutamicum*, its editing efficiency remains low [15, 21]. Moreover, without the introduction of recombinases, it is hard to accomplish gene insertion into the *C. glutamicum* genome. Figure 1 indicates that RecET facilitates the integration of DNA fragments into the genome. In our CRISPR–Cpf1 system, two compatible plasmids were used (Additional file 1: Fig. S3): the plasmid pEC-RecET enabling constitutive expression of RecET in *C. glutamicum*, and a pXM-Cpf1-crRNA-donor derivative, carrying the *Fn*Cpf1 expression cassette, crRNA expression cassette, and donor DNA template for integration into the genome. After the introduction of the pXM-Cpf1-crRNA-donor plasmid, two events occurred, namely the Cpf1-induced DSB and the RecET-promoted HR between the host genome and dsDNA donor molecules, which ultimately produced genome-edited colonies.

**Fig. 2:**
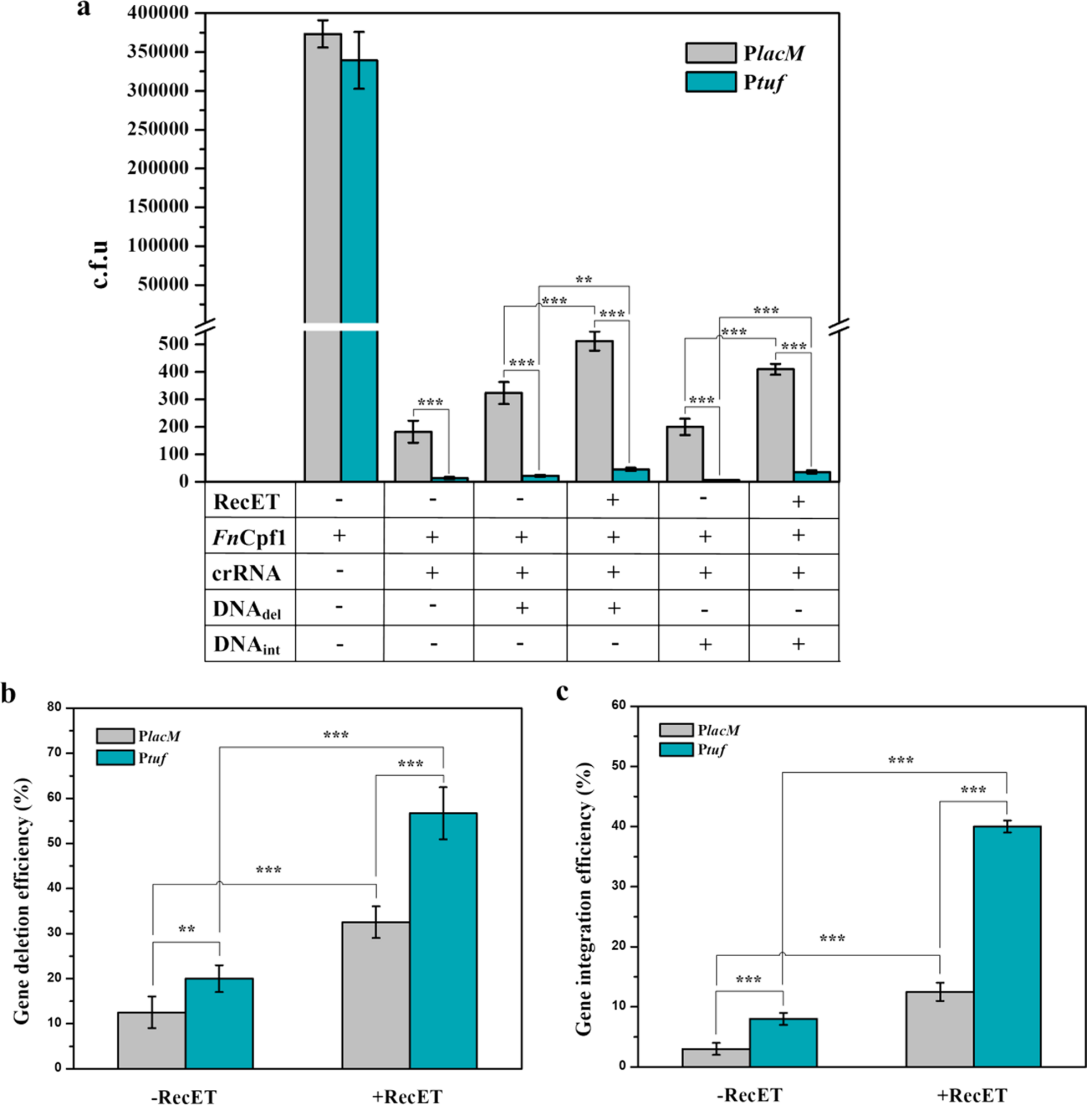
Gene deletion and insertion using the CRISPR–Cpf1 system, and the effects of RecET expression and promoters that drive *Fn*Cpf1 expression are represented. **a** Colonies formed under different conditions. When *Fn*Cpf1 and crRNA were both expressed, the number of colonies was several orders of magnitude lower than that observed in the control, indicating that the Cpf1–crRNA complex is functional in *C. glutamicum*. The expression of RecET significantly increased the number of colonies, probably by improving the homologous recombination (HR) activity. P_tuf_-driven expression of *Fn*Cpf1 decreased the number of colonies compared to that in the P_lacM_-*Fn*Cpf1 expressing cassette. **b** Gene deletion efficiencies (deletion of 534 bp from *Cg*Del); **c** Gene integration efficiencies (insertion of 528 bp into *Cg*Int). The introduction of RecET and the P_tuf_ promoter significantly enhanced gene deletion and integration. Experiments were performed in triplicates. Values are presented as mean ± SD. **P < 0.01, *** *P* < 0.001

For *Fn*Cpf1 expression, two promoters, P_lacM_ [15] and P_tuf_, were used for comparison. The crRNA was expressed under the control of the P_j23119_ promoter [13]. The upstream and downstream homologous fragments for the donor DNA were both approximately 500 bp long (Additional file 1: Fig. S1). To confirm the function of the Cpf1–crRNA complex, the plasmids pXM-plCpf1-crRNA (P_lacM_-*Fncpf1*) and pXM-ptCpf1-crRNA (P_tuf_-*Fncpf1*) were introduced into the model strain *Cg*Del harboring pECspec (the control plasmid of pEC-RecET), with electroporation of the non-crRNA plasmids pXM-plCpf1 and pXM-ptCpf1, respectively, as controls. It was observed that colonies grown on agar plates were several orders of magnitude lower in number than those of their respective control groups, indicating that the Cpf1–crRNA complex efficiently cleaved the *C. glutamicum* genome. Furthermore, when *Fn*Cpf1 was expressed under the strong promoter P_tuf_, transformant survival was approximately 13-fold less than that under the P_lacM_ promoter (Fig. 2a). Next, we introduced two plasmids pXM-plCpf1-crRNA-donor_del_ and pXM-ptCpf1-crRNA-donor_del_, separately into *Cg*Del harboring pECsepc or pEC-RecET. In the presence of donor DNA templates that could be integrated into the *Cg*Del chromosome, the survival rates were markedly improved (Fig. 2a), implying that the integration of donor fragments might prevent Cpf1 cleavage in the genome. We then randomly selected colonies and inoculated them onto agar plates supplemented with kanamycin, and some colonies indeed grew. Subsequent colony PCR and DNA sequencing analyses confirmed the deletion of the *xylA*_Eco_* DNA fragment. As expected, the presence of RecET in competent cells dramatically increased transformant survival (Fig. 2a) and improved gene deletion efficiency (Fig. 2b). The P_tuf_-driven expression of *Fn*Cpf1 resulted in fewer transformants, but it increased the gene deletion efficiency as compared to the P_lacM_-driven expression of *Fn*Cpf1. Next, we attempted to insert a 528 bp DNA fragment into the genome of *Cg*Int (Additional file 1: Fig. S1b). To do this, two plasmids, pXM-plCpf1-crRNA-donor_int_ and pXM-ptCpf1-crRNA-donor_int_, were employed. As observed before, the introduction of RecET considerably increased the number of transformants (Fig. 2a) and gene integration efficiency (Fig. 2c); the high expression of *Fn*Cpf1 was also beneficial for gene insertion. Thus, our one-step two-plasmid-based CRISPR–Cpf1 system enabled gene deletions and insertions with high efficiency. The efficiencies of an approximately 500 bp deletion and 500 bp insertion achieved approximately 57% and 40%, respectively.

In addition, we attempted gene deletion and insertion using linear DNA as the donor templates. Briefly, the plasmid pXM-ptCpf1-crRNA*,* along with the linear donor_del_ or donor_int_ templates, was electroporated into *Cg*Del or *Cg*Int harboring pEC-RecET. The results showed an average deletion efficiency of approximately 22% for the deletion of the 534 bp DNA fragment (Additional file 1: Fig. S4). However, we could not obtain any colonies with chromosomal gene insertions. To demonstrate the potential application of our one-step CRISPR-Cpf1 genome editing system in metabolic engineering, we modified *C. glutamicum* to produce alanine. First, *ldhA* was deleted using a linear DNA fragment as the donor, with a deletion efficiency of 5%. Second, the *Bacillus subtilis-*derived *alaD* gene [32] under the P_sod_ promoter was integrated using the plasmid pXM-ptCpf1-crRNA_*cg1890*_-*alaD*_int_, with an integration efficiency of 33%. After plasmid curing, 8.5 g/L alanine was produced through shake-flask fermentation (Additional file 1: Fig. S5).

### Establishment of a two-step CRISPR-Cpf1 genome editing technology

The efficiencies of our one-step editing system are acceptable but not sufficiently high. Surprisingly, during the development of this system, the RecET-mediated HR between the donor DNA and the genome was observed to be extraordinarily high (Fig. 1). In contrast, fewer colonies grew when the Cpf1–crRNA complex was expressed with the donor DNA provided. Additionally, the colonies were also high when only Cpf1 was expressed (in the absence of the Cpf1–crRNA cutting system), and therefore, we presumed that the crRNA-guided Cpf1 cleavage at the targeted genomic locus interfered with HR in *C. glutamicum*. This observation is against an earlier report that CRISPR/Cas9-mediated DSBs enhance HR efficiency in *E. coli* [33]. Based on our findings, we decided to investigate these two events separately by attempting to develop a two-step genome editing technology that harnesses the DSBs created by CRISPR/Cpf1 as a counterselection.

**Fig. 3:**
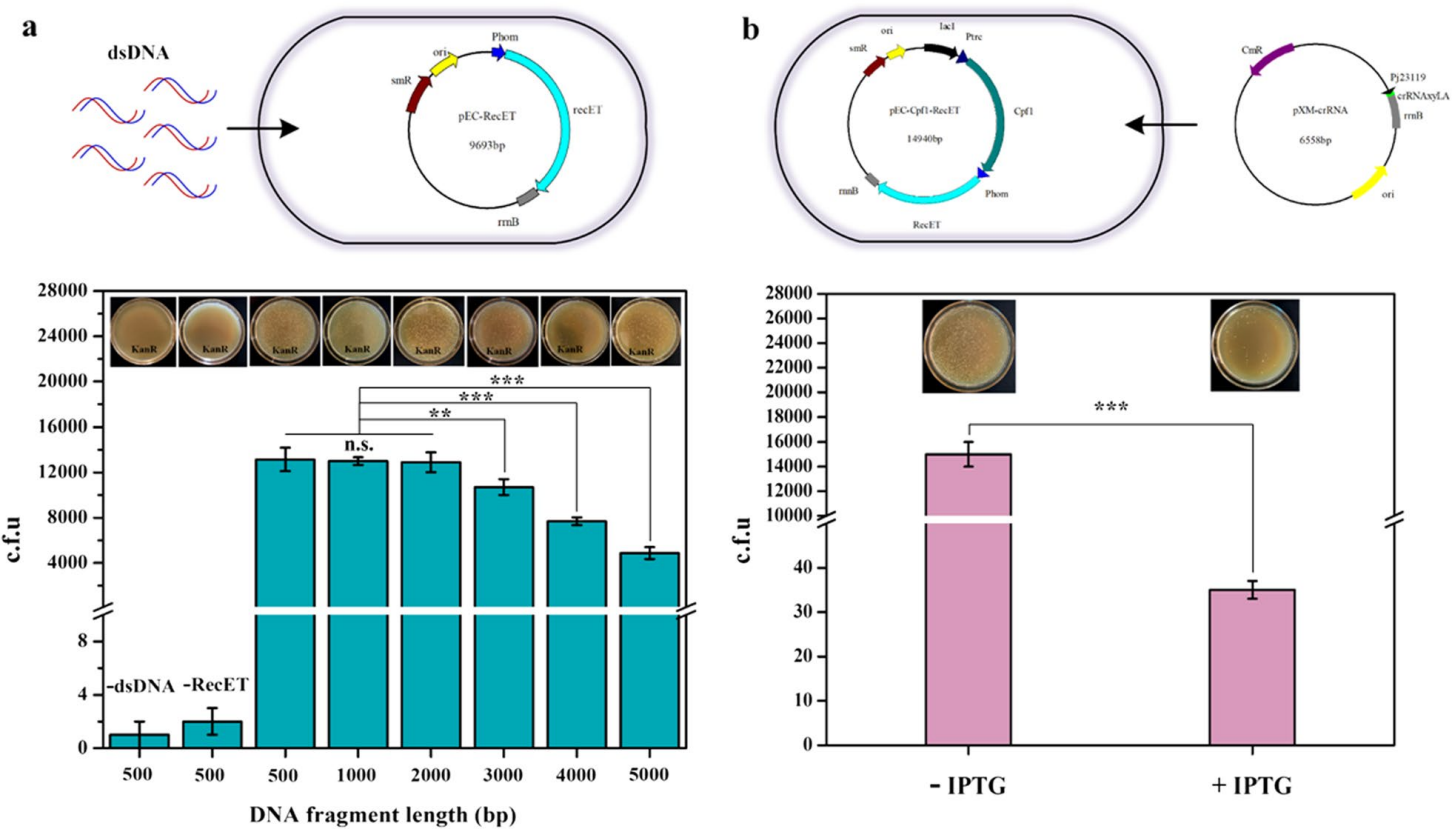
RecET-mediated HR and IPTG-inducible Cpf1 genomic cleavage, potentially applicable for the development of a two-step CRISPR–Cpf1 system. **a** Linear templates with different lengths are used for integration, and kanamycin-resistant colonies represent the HR activity. **b** A decrease in the number of colonies after IPTG addition represents a strong inducible effect. The colonies were counted or calculated based on dilution. Experiments were performed in triplicates. Values are presented as mean ± SD. **P < 0.01, *** *P* < 0.001; n.s., not significant

Although a 500 bp donor being mediated by RecET, could be integrated with high efficiency, we were particularly interested in the integration of large donor DNAs. To this end, we constructed a series of donor DNA fragments of different lengths (500–5000 bp, Additional file 1: Fig. S1b). These DNA fragments were electroporated into *Cg*Int cells harboring pEC-RecET. With kanamycin selection, there were approximately 1.3 × 10^4^ colonies per plate for 500–2000 bp fragments. For fragments of more than 2000 bp, the number of colonies gradually decreased with the increasing length of DNA. However, it still exceeded 4.8 × 10^3^ even when the DNA length reached 5000 bp (Fig. 3a). Furthermore, we placed these fragments separately within the plasmid and examined their integration efficiencies using recombinase RecET. The integration of plasmid-borne donor DNA by HR was more efficient than that of linear DNA.

Next, we sought to establish an appropriate inducible system for *Fn*Cpf1 expression. Inducible promoters, such as IPTG-inducible P_tac_ and propionate-inducible P_prpD2_ [17–19] are often used for the expression of toxic Cas9 in *C. glutamicum*. In this study, we compared the IPTG-inducible P_tac_, arabinose-inducible P_BAD_ [31] and propionate-inducible P_prpD2_ in terms of *Fn*Cpf1 expression. The propionate induction results were unsatisfactory (data not shown). For the IPTG and arabinose-inducible groups, very few colonies appeared on agar plates supplemented with their corresponding inducer compared with those grown without inducer, indicating the strictly inducible expression of *Fn*Cpf1. The ratio of colonies formed with and without an inducer was lower for the IPTG-inducible expression of *Fn*Cpf1, compared with that for the arabinose-inducible system (Additional file 1: Fig. S6). Based on these findings, we proposed a two-plasmid-based inducible Cpf1–crRNA cleavage system (Fig. 3b). The transformation efficiency of pXM-crRNA into *Cg*Del harboring pEC-Cpf1-RecET without IPTG addition reached approximately 1.5 × 10^4^ colonies per plate. More importantly, only a few dozens of colonies escaped the cleavage of the Cpf1-crRNA complex because of IPTG induction. Additionally, this two-plasmid system allows the construction of large DNA fragments in pXMJ19 derivatives, which is a prerequisite for the genomic insertion of larger genes.

Efficient plasmid curing is essential for the iterative gene manipulation. Therefore, we investigated the curing of the two plasmids, the pXMJ19 and pEC-XK99E series. The pXMJ19ts with a temperature-sensitive replication origin [34] has often been applied to *C. glutamicum* genome editing [15, 20]. We found that although the plasmid curing efficiency was 96% at 37 °C, the transformation efficiency, even at 32 °C, decreased drastically (Additional file 1: Fig. S7a), indicating that temperature-sensitive plasmids were not suitable for the two-plasmid system. We used *sacB*-mediated sucrose selection for plasmid curing, and the efficiency observed was 100% after one liquid culture with sucrose (Additional file 1: Fig. S7b). For the curing of pEC-XK99E derivatives, the gene encoding distribution protein Per1 [35] was deleted to facilitate plasmid curing as reported by Jiang et al [15]. The curing efficiency was approximately 90% (Additional file 1: Fig. S7c). Herein, we also demonstrated that plasmid curing for pXMJ19 and pEC-XK99E derivatives was rather difficult after culturing without antibiotics. In some studies, the continuous passage was reportedly utilized, which is a time-consuming and impractical method.

**Fig. 4:**
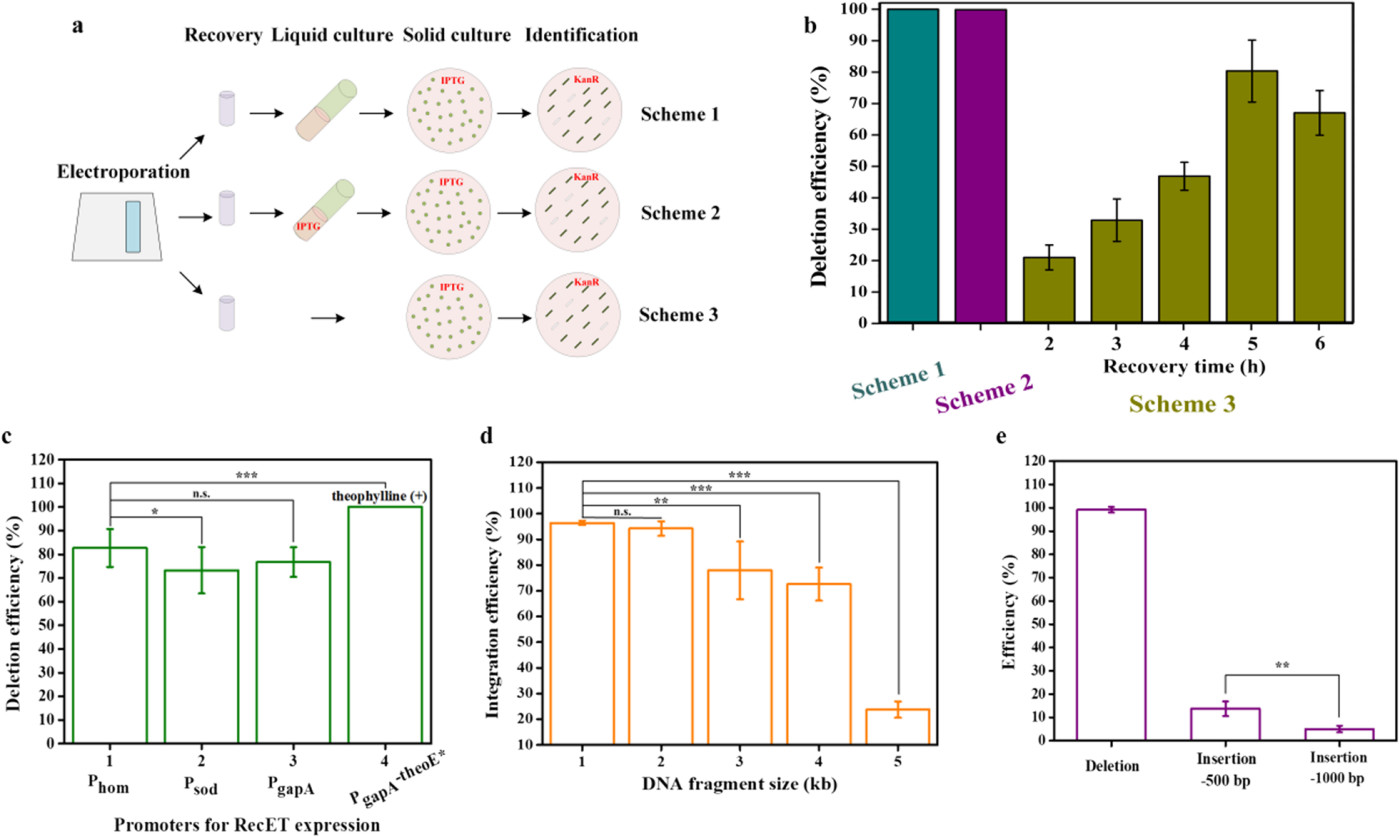
Optimization of the two-step RAPID CRISPR–Cpf1 system. **a** Different operating procedures for the RAPID system, scheme 1—the transformants are cultured in liquid medium without IPTG induction after 2 h recovery, prior to inoculation onto solid medium supplemented with IPTG. scheme 2—is the same as scheme 1, except that IPTG is added to the liquid culture. scheme 3—the transformants are recovered for a different time duration and directly spread onto a solid medium supplemented with IPTG. **b** Gene deletion efficiencies for different operating schemes. **c** Promoter optimization for RecET expression. **d** Integration efficiencies for fragments of different sizes. **e** Gene deletion and integration efficiencies using linear DNA template. Experiments were performed in triplicates. Values are presented as mean ± SD. *P < 0.05, **P < 0.01, *** *P* < 0.001; n.s., not significant

With a highly efficient HR system and a strictly inducible Cpf1 cleavage system in *C. glutamicum*, we proposed the use of a RecombinAtion Prior to Induced Double-strand-break (RAPID) genome editing technology. In this method, the expression of RecET in competent cells might result in efficient HR of donor DNAs without the interference of Cpf1–crRNA genomic cleavage. Subsequently, the inducible expression of *Fn*Cpf1 would trigger the DSBs, eliminating unedited cells. To investigate the feasibility of the RAPID technology, we took the deletion of *xylA*_Eco_* from the genome of *Cg*Del as an example. The plasmid pXM*sacB*-crRNA-donor_del_ was introduced into competent cells harboring pECΔ*per1*-Cpf1-RecET. With the notion of “recombination prior to induced DSB” we first proposed an operating procedure with the liquid culture after electroporation and recovery, prior to spreading the culture onto a solid medium supplemented with IPTG (Fig. 4a, Scheme 1). Under the kanamycin resistance, all the tested colonies were verified to be with correct gene deletion (Fig. 4b). We also conducted liquid culture with IPTG addition (Fig. 4a, Scheme 2), and the gene deletion efficiency reached 100%, which implied that recombination between the donor DNA and chromosome might occur during the recovery process. Therefore, we skipped the liquid culture procedure and prolonged the recovery time from the standard duration of 2 h to 6 h (Fig. 4a, Scheme 3). The results indicated that a 5 h recovery resulted in the highest gene deletion efficiency, exceeding 80% (Fig. 4b, Additional file 1: Fig. S8a). This simplifies the operating procedure of the RAPID system without much discount of the editing efficiency.

As the efficiency of HR was crucial in our genome editing system, we explored different promoters, P_sod_, P_gapA_, and P_gapA_ with a theophylline riboswitch [36] for RecET expression. For the P_gapA_-theoE*-driven expression of RecET, 1 mM theophylline was added to the culture to prepare competent cells. When RecET was expressed under the control of P_sod_ and P_gapA_, the gene deletion efficiencies were lower than that of P_hom_-expressed RecET. In the P_gapA_-theoE* group, the gene deletion efficiency reached 100% (Fig. 4c). With the successful introduction of the theophylline-induced expression of RecET, recovery times ranging between 2 and 6 h resulted in an almost 100% gene deletion. We did not attempt the deletion of longer fragments, as deletion of a DNA fragment < 1 kb is sufficient for gene inactivation in most practical applications.

We then investigated the integration of DNA fragments of lengths of 1–5 kb, into the *Cg*Int genome. After electroporation, the cells were recovered for 5 h, then spread onto the solid medium supplemented with IPTG. The integration efficiency of the 1 kb DNA fragment reached 96%, which gradually decreased with the increasing length of DNA fragments. Surprisingly, a 4 kb DNA was integrated into the genome with an efficiency higher than 72%, and a 5 kb DNA fragment could be successfully integrated with 24% efficiency (Fig. 4d, Additional file 1: Fig. S8b).

**Fig. 5:**
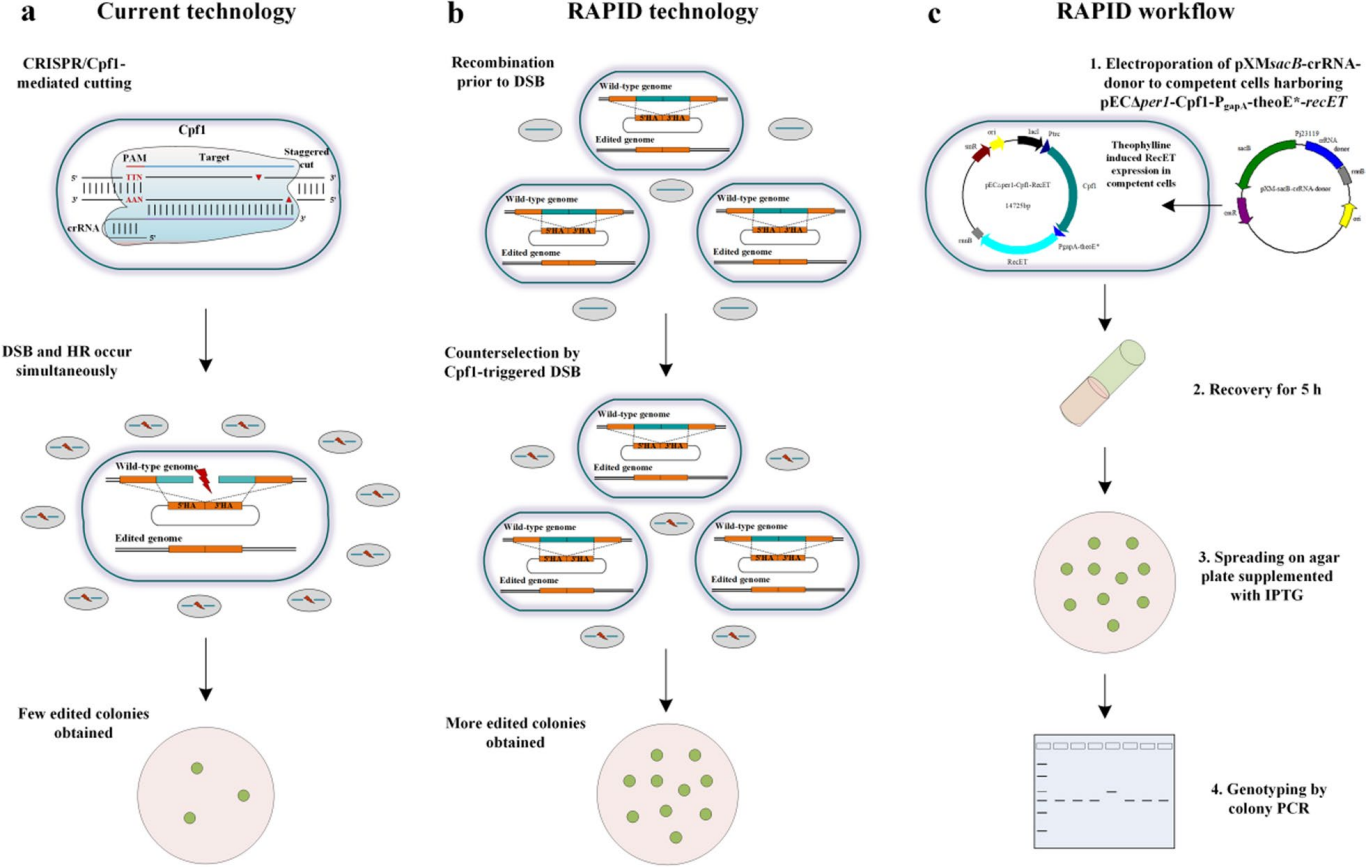
Overview of the RAPID genome editing system. **a** Principle of the currently available one-step CRISPR technologies. **b** Principle of the RAPID technology, wherein CRISPR-crRNA is used as a counterselection, which significantly increases the number of edited colonies owing to the high-efficiency HR mediated by RecET. **c** Workflow of the RAPID technology, wherein the transformation of linear donor fragments and pXM-*sacB*-crRNA can be used for gene deletion. The whole process is simplified and comparable to that of the one-step CRISPR genome editing system

In addition, gene deletion (*Cg*Del) and insertion (*Cg*Int) were investigated using linear DNA fragments as the donor templates (Fig. 4e). The efficiency of deleting 534 bp *xylA*_Eco_* was almost 100%. The efficiencies for the integration of the 500 and 1000 bp DNA fragments were approximately 14% and 5%, respectively. However, fragments larger than 1000 bp (i.e., 2, 3, 4, and 5 kb) could not be inserted into the genome of *Cg*Int. Therefore, for practical concerns, we had to use a plasmid-borne DNA template for gene insertion, and a linear template for gene deletion. In summary, we established a highly efficient CRISPR-Cpf1 genome editing technology including a two-step process as follows: RecET-assisted recombination and Cpf1/crRNA-induced DSB as a counter selection. Our RAPID technology allows efficient recombination before the induction of *Fn*Cpf1, and thus enables more edited colonies to survive. This method may enable recombination and ensure that bacterial chromosomes are fully segregated before applying the negative selection [37]. Although two steps are involved, the operating procedure of RAPID is quite simple (Fig. 5).

### Characterization of RAPID methodology

The CRISPR-based genome editing technology that harnessed the DSBs created by CRISPR/Cpf1 as a counterselection was developed, based on the finding that Cpf1 cleavage disrupted RecET-mediated HR of the donor template into the genome. In most reported studies, exogenous recombinases were not included to ameliorate the low HR activity of *C. glutamicum*, resulting in few transformants and low gene deletion/insertion efficiencies. To accomplish genome editing, a low HR often requires highly efficient genomic cleavage, which cannot be achieved without the optimization of the gRNAs/crRNAs targeting corresponding genomic protospacers. Previously, Zhao et al. established a RecET-assisted CRISPR-Cpf1 system in *C. glutamcium*, which could accomplish highly efficient gene deletion owing to the expression of RecET [38]. However, the transformants were few in the circumstance of simultaneous Cpf1-triggered cutting and RecET-assisted recombination. To cope with the low recombination and few transformants, Zhang et al. applied CRISPR/Cpf1 technology to assist in the selection of edited second crossover strains after pk18*mobsacB*-mediated single recombination [22], which was complicated and time-consuming. In the RAPID system, a high dsDNA recombination efficiency was achieved owing to RecET expression before the inducible expression of *Fn*Cpf1. Thus, the linear template is sufficient to achieve efficient gene editing. The RAPID system ensures more robust and stable genomic manipulations that can be identified from abundant transformants.

Because Cas9 is toxic to *C. glutamicum* and is easily mutated, it is not feasible to introduce it into competent cells [18]. Cas9 expression does not allow large donor DNA integration in one plasmid. Yao et al. reported insertions using large fragments or genes (> 3 kb) as unsuitable because of the plasmid size [24]. To overcome these difficulties, *cas9* and *recET* genes were integrated into the genome and removed using the suicide plasmid-based method [20, 23]. Herein, two compatible plasmids were used in the RAPID genome editing system, and the non-toxic *Fn*Cpf1 and RecET recombineering system from the *E. coli* Rac prophage were applied. Using an inducible expression cassette, both systems were integrated into one plasmid of the competent cells. This increased the loading capacity of another plasmid, providing the possibility of large fragment insertion. A large DNA fragment (e.g., 4 kb long) can effectively integrate into the genome with a plasmid-borne template. The size of a bacterial gene is normally less than 3 kb, and the segment-divided approach, as previously reported for use in *E. coli* [39], may be useful for the integration of a large operon within the bacterial genome.

Owing to the transcribed RNA-processing capability of Cpf1 [13], we constructed pXM-*sacB*-crRNA-donor plasmid series harboring a crRNA-expressing cassette without an immediate terminator. The results revealed that the crRNA expressed in this manner could be processed by Cpf1 and sufficiently triggered the cleavage of the genomic target. This finding enabled simplified plasmid construction, which was attributed to the omission of the amplification of a fragment containing the crRNA cassette. To this end, we placed P_j23119_ and the 19 bp direct repeat in the starting plasmid, and the 24 bp spacer sequence was designed by utilizing the primer used for amplifying the donor DNA. When the donor fragment was ligated into the linear plasmid, the promoter P_j23119_ transcribed the complete crRNA. When a linear DNA fragment was used as the donor, the synthesized base pair of oligonucleotides were annealed and ligated directly into the plasmid.

In addition, the RAPID system enables the iterative genome engineering of *C. glutamicum*. A simple and efficient plasmid-curing method is required for the iterative operations. Herein, the SacB-triggered cell lethality was proven to be a good choice for plasmid curing. For each cycle of iterative genome editing using the RAPID methodology, only the pXMJ19-derived crRNA-expressing plasmid needs to be cured. To obtain a plasmid-free strain, the pECΔ*per1*-Cpf1-recET plasmid can be readily cured by culturing without antibiotic supplementation.

### Application of RAPID for metabolically engineering a D-PA producer

**Fig. 6:**
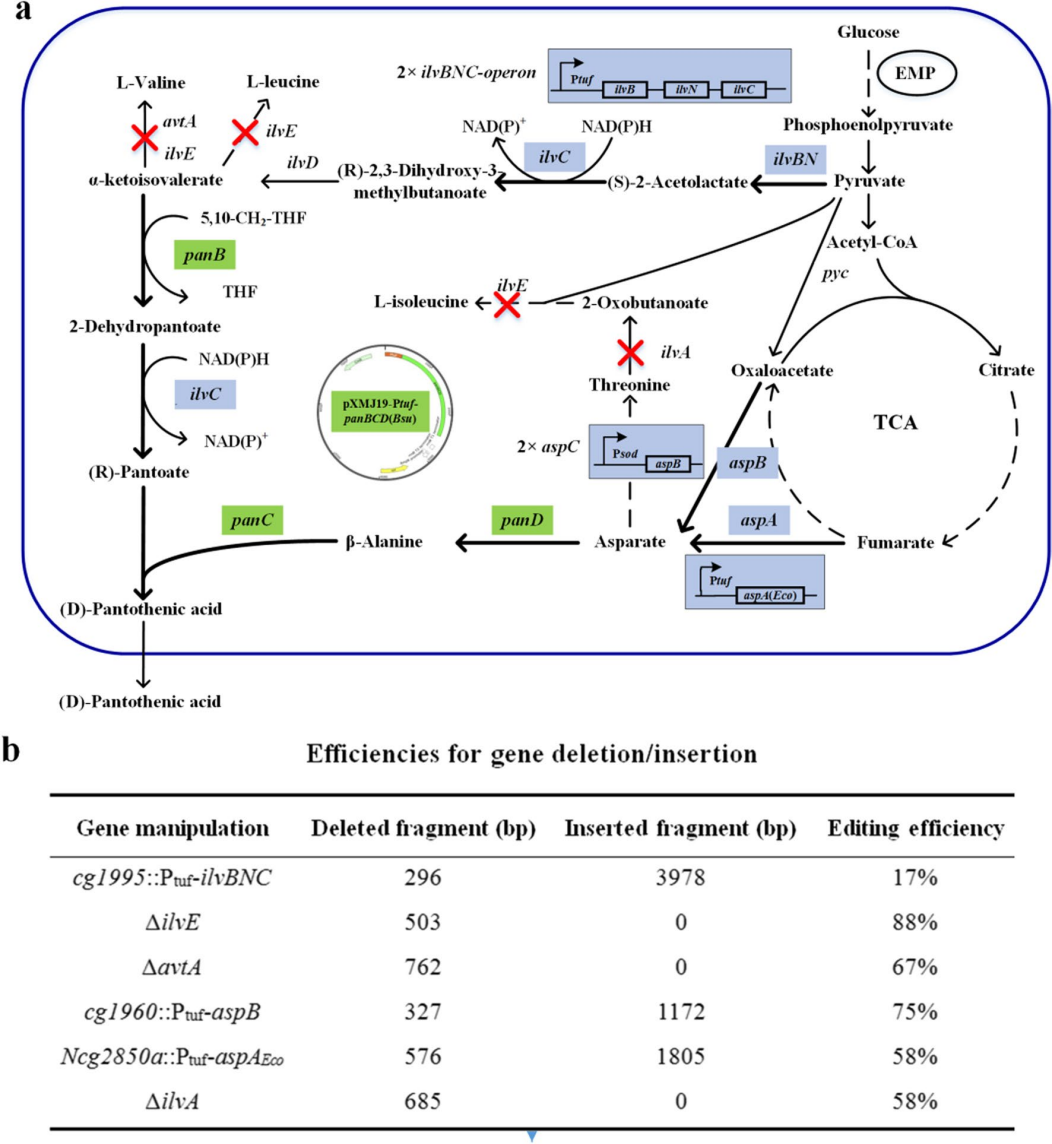
Biosynthetic pathways of D-PA in *C. glutamicum* and the strategies for constructing the D-PA producing strain, and efficiencies for the corresponding genome editing using RAPID. **a** The red X represents the gene that was deleted, the blue boxes represent genes that were overexpressed by genomic integration using a strong promoter, and the green boxes represent genes that were overexpressed in a plasmid: *ilvBN* (acetohydroxy acid synthase), *ilvC* (ketol-acid reductoisomerase and ketopantoate reductase), *ilvE* (branched-chain-amino-acid aminotransferase), *avtA* (valine-pyruvate aminotransferase), *aspB* (aspartate aminotransferase), *aspA* (aspartate ammonia lyase from *E. coli*), *ilvA* (threonine dehydratase), *panB* (α-ketoisovalerate hydroxymethyltransferase from *Bacillus subtilis*), *panC* (pantothenate synthetase from *B. subtilis*), and *panD* (aspartate 1-decarboxylase from *B. subtilis*). **b** The efficiencies of the above-mentioned gene deletions and insertions. A 500‒800 bp fragment was normally deleted for gene inactivation, and a small fragment (here less than 600 bp) was replaced by the inserted gene cassette

The CRISPR–Cpf1-mediated RAPID genome editing technology significantly enhanced our ability to perform genomic manipulation in *C. glutamicum*. To demonstrate its potential application in metabolic engineering, we used RAPID to modulate the *C. glutamicum* genome to overproduce D-PA. D-PA is biosynthesized by the condensation of (R)-pantoate and β-alanine, and its synthetic pathway can be divided into two branches: (R)-pantoate synthetic pathway and β-alanine synthetic pathway (Fig. 6a). (R)-Pantoate is synthesized from α-ketoisovalerate, the direct precursor of l-valine, using α-ketoisovalerate hydroxymethyltransferase and 2-dehydropantoate 2-reductase, which are encoded by *panB* and *ilvC* (in *C. glutamicum*), respectively. β-Alanine is decarboxylated from L-aspartate by aspartate 1-decarboxylase (encoded by *panD*).

In this study, wild type *C. glutamicum* ATCC 13,032 was used as the starting strain. First, to enhance the synthetic pathway to α-ketoisovalerate, the *ilvBNC* operon from *C. glutamicum* XV (NCBI accession NZ_CP018175.1), a high L-valine producer generated by mutagenesis, was chromosomally integrated at the *cg1995* locus under the control of the P_tuf_ promoter. The *ilvE* and *avtA* genes encoding aminotransferases for the synthesis of branched-chain amino acids were then deleted, reducing the competition of α-ketoisovalerate consumption. The resulting strain was named Pan-1. Subsequently, *aspB* (*cg0294*, encoding aspartate aminotransferase) and *aspA* from *E. coli* (encoding aspartate ammonia lyase) were successively integrated into the *C. glutamicum* genome at the loci *cg1960* and *Ncgl2850a*, respectively, to obtain the strains Pan-2 and Pan-3. As α-ketobutyrate can replace α-ketoisovalerate as a substrate for ketopantoate hydroxymethyltransferase [40], we deleted *ilvA* to block its synthesis, generating the strain Pan-4. Gene deletions were performed using linear donor templates. The efficiencies of these genomic manipulations are presented in Fig. 6b, and the results of the colony PCR identification are shown in Additional file 1: Fig. S9. The *panBCD* operon from *B. subtilis* was cloned and expressed in pXtuf [41]. The resulting plasmid, pXtuf-*panBCD*_*Bsu*_, was introduced into these strains to generate the D-PA producers.

**Fig. 7:**
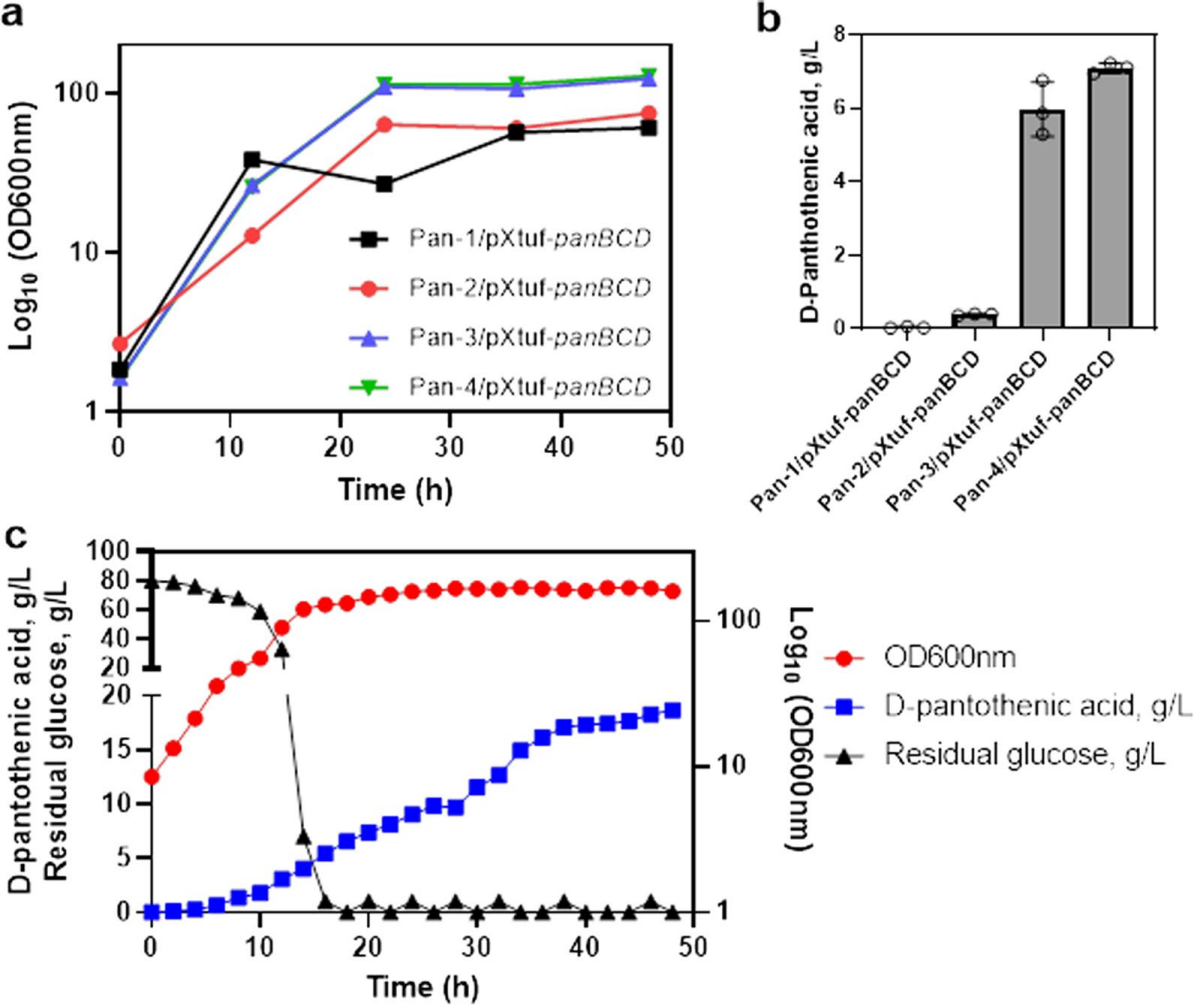
Production of D-PA using shake-flask fermentation and in a 5 L fermenter. **a** The biomass (optical density (OD) at 600 nm) of these strains using shake-flask fermentation. **b** The titers of D-PA in shake-flask fermentation. **c** The biomass, D-PA titer and residual glucose of strain Pan-4/pXtuf-*panBCD* in a 5 L fermenter. The shake-flask fermentation was performed in triplicate, and values are presented as mean ± SD

Shake flask fermentation of these strains was performed (Fig. 7a, b). The growth of Pan-1 was observed to be reduced, indicating that the accumulation of α-ketoisovalerate may be detrimental to bacterial growth. Surprisingly, this strain, harboring the plasmid pXtuf-*panBCD*_*Bsu*_, could not produce D-PA. A plausible reason could have been an insufficient amount of l-aspartate, which is required for β-alanine synthesis. Thus, we attempted to enhance the l-aspartate levels by overexpressing *aspB* and *aspA*. Indeed, Pan-2/pXtuf-*panBCD*_*Bsu*_ cells overexpressing *aspB* produced 0.38 g/L D-PA. Interestingly, the expression of *aspA*_*Eco*_ significantly increased cell growth and D-PA production (5.57 g/L). We speculated that the cellular metabolic pathway of Pan-2 might be imbalanced since the important intermediate metabolite pyruvate was directed to the α-ketoisovalerate and l-aspartate synthesis pathway. After the expression of *aspA*_*Eco*_, the reverse reaction from l-aspartate to fumarate [42] might effectively maintain the TCA cycle, improving cell robustness. Finally, the strain Pan-4/pXtuf-*panBCD*_*Bsu*_ with *ilvA* deletion produced 7.09 g/L D-PA, suggesting the disruption of α-ketobutyrate synthesis is an effective strategy. We also attempted to produce D-PA through the genomic integration of *panBCD*_*Bsu*_; however, the results were unsatisfactory, as discussed in a previous study [41]. Moreover, using glucose as main carbon source without β-alanine addition, Pan-4/pXtuf-*panBCD*_*Bsu*_ achieved the highest D-PA titer of 18.62 g/L (Fig. 7c) in a 5 L fermenter, which is much higher than these reported titers in *C. glutamicum* of less than 2 g/L [43], and in *E. coli* of 12.33 g/L [44]. Thus, the final *C. glutamicum* strain produced the highest titer of D-PA obtained by microbial de novo synthesis using glucose as the main carbon source.

## Conclusions

We developed a RecET-assisted CRISPR–Cpf1 genome editing technology for *C. glutamicum* that harnessed CRISPR-induced DSBs as a counterselection. This method was named after RecombinAtion Prior to Induced Double-strand-break (RAPID) genome editing, which enabled fast gene manipulations with high efficiency. The expression of *Fn*Cpf1 and RecET in competent cells and the transcribed RNA-processing ability of Cpf1 simplify the construction of a plasmid expressing the crRNA and make harboring a large DNA donor possible. This simplified procedure and iterative operation ability further guarantee a fast and efficient strain construction. Moreover, the D-PA *C. glutamicum* producer constructed by RAPID was demonstrated to achieve the highest reported titer of 18.62 g/L from glucose as the main carbon source without β-alanine addition. Taken together, the RAPID method is of great importance to *C. glutamicum* genome editing in terms of its practical applications, which also guides the development of CRISPR genome editing tools for other microorganisms.

## Methods

### Stains and media

All bacterial strains used in this study are listed in Additional file 1: Table S1. *E. coli* DH5α was used for general cloning work. *C. glutamicum* ATCC 13,032 was used for the construction of alanine and D-PA producing strains. *C. glutamicum rpsL*^K43T^ strain [47] was used for developing CRISPR/Cpf1 genome editing systems. *E. coli* DH5α was grown aerobically in Luria–Bertani (LB) medium (10 g/L tryptone, 5 g/L yeast extract, and 10 g/L NaCl) at 37 °C, with shaking at 200 rpm. *C. glutamicum* strains were routinely cultivated in brain heart infusion-supplemented (BHIS) medium (37 g/L BHIS medium and 91 g/L sorbitol) at 30 °C, with shaking at 200 rpm. Where necessary, antibiotics were added at the following concentrations: 50 μg/mL kanamycin, 30 μg/mL chloramphenicol, and 50 μg/mL spectinomycin for *E. coli* and 10 μg/mL kanamycin, 10 μg/mL chloramphenicol, 200 μg/mL spectinomycin and 50 μg/mL streptomycin for *C. glutamicum*. Agar was added at 15 g/L for solid plates. *E. coli* DH5α, for plasmid construction, was transformed by the CaCl_2_-mediated method [45]. All *C. glutamicum* strains were transformed by electroporation. BHIS medium was used for the preparation of *C. glutamicum* electrocompetent cells. All strains were kept as glycerol stocks prepared in LB or BHIS broth containing 16% glycerol at − 80 °C.

For the production of D-PA, the agar-slant medium contained (per liter) 10 g of tryptone, 10 g of beef extract, 5 g of yeast extract, 25 mL of corn steep liquor, 1 g of KH_2_PO_4_, 0.5 g of MgSO_4_·7H_2_O, 2.5 g of NaCl, and 25 g of agar, with pH adjusted to 7.0–7.2. The seed culture medium in a 500 mL flask or the 5 L bioreactor included (per liter): glucose 40 g, yeast extract 3 g, tryptone 2 g, corn steep liquor 15 mL, (NH_4_)_2_SO_4_ 5 g, KH_2_PO_4_ 2.5 g, MgSO_4_·7H_2_O 1.6 g, FeSO_4_·7H_2_O 20 mg, MnSO_4_·H_2_O 20 mg, VB_1_ 0.3 mg, V_H_ 0.1 mg, L-valine 1 g, L-leucine 1 g, L-isoleucine 1 g, l-glutamic acid 5 g, and citric acid 2 g (pH 7.0–7.2). D-PA fermentation medium included (per liter): glucose 80 g, yeast extract 3 g, tryptone 2 g, corn steep liquor 40 mL, soybean protein hydrolysate 10 mL, (NH_4_)_2_SO_4_ 5 g, KH_2_PO_4_ 2.5 g, MgSO_4_·7H_2_O 1.6 g, FeSO_4_·7H_2_O 20 mg, MnSO_4_·H_2_O 20 mg, VB_1_ 0.3 mg, V_H_ 0.1 mg, l-valine 1 g, l-leucine 1 g, l-isoleucine 1 g, L-glutamic acid 5 g, and citric acid 2 g (pH 7.0–7.2).

### Plasmid and strain construction

All plasmids used in this study are listed in Additional file 1: Table S1. All primers and the oligonucleotide used in the study are listed in Additional file 1: Table S2. Chromosomal DNA was extracted using a bacterial genomic DNA extraction LRC021 kit (Lanry Bio, Tianjin, China). Plasmid and DNA fragment extractions were conducted using E.A.N.A. kits (Omega Bio-Tek). PCRs for fragment amplification and colony PCR used PrimeSTAR HS DNA Polymerase (Takara) and 2 × Rapid Taq Master Mix (Vazyme Biotech, Nanjing, China), respectively. DNA fragment ligation was performed by overlap extension PCR. The ligation of the plasmid backbone (linearized by restriction endonucleases) and DNA fragments was performed through HR by using a ClonExpress II One Step Cloning Kit (Vazyme). The detailed information for plasmid construction and linear donor template preparation is provided in Supplementary materials.

To obtain D-PA producing strains, the wild-type *C. glutamicum* ATCC 13032 was used as the starting strain. Iterative genome editing was performed according to the procedures described below. For gene deletion, we used a pXM*sacB*-crRNA derivative that expressed the crRNA targeting the corresponding genomic locus, and the linear donor template was electroporated simultaneously into competent cells harboring pECΔ*per1*-Cpf1-P_gapA_-theoE*-RecET. For gene insertion, a pXM*sacB*-crRNA-donor_int_ derivative containing the corresponding crRNA cassette and donor DNA template was electroporated. The edited strains were identified by colony PCR and subsequent DNA sequencing.

### Iterative RAPID genome editing procedure

The Cpf1- and RecET-expressing plasmid pECΔ*per1*-Cpf1-P_gapA_-theoE*-RecET was introduced into *C. glutamicum* by electroporation. Electrocompetent cells were generated as previously described [46]. For the preparation of competent cells of strains harboring pECΔ*per1*-Cpf1-P_gapA_-theoE*-RecET, 1 mM theophylline was added to the culture to induce RecET expression. Electroporation was performed using the Eppendorf Eporator system set to 1850 V. In the plasmid-borne template system, approximately 500 ng of a pXM*sacB*-crRNA-donor derivative was used for each electroporation. A linear template was sufficient for gene deletion. In this system, appropriately 500 ng of a pXM*sacB*-crRNA derivative and 1 μg of the linear donor were mixed to co-transform into competent cells. The transformed cells were immediately transferred into 1 mL of pre-warmed (46 °C) BHIS medium and incubated for 6 min at 46 °C without shaking. Subsequently, *C. glutamicum* cells were recovered for 2–5 h at 30 °C with shaking at 200 rpm. Finally, cells were spread onto BHIS agar plates supplemented with 10 μg/mL chloramphenicol and 200 µg/mL of spectinomycin and incubated at 32 °C; 0.1 mM isopropyl-β-d-thiogalactopyranoside (IPTG) was also supplemented in the agar medium to induce *Fn*Cpf1 expression. Single colonies were checked by colony PCR and subsequently confirmed by DNA sequencing.

Edited colonies containing plasmids of pECΔ*per1*-Cpf1-P_gapA_-theoE*-RecET and a pXM*sacB*-crRNA derivative were incubated in BHIS medium containing spectinomycin and 1.5% (w/v) sucrose for 6–8 h or overnight at 30 °C. Cultures were streaked and colonies were tested for chloramphenicol sensitivity. After curing the pXM*sacB*-crRNA series, colonies were grown to the mid-log phase to prepare electrocompetent cells for the next round of editing. After completing all the genomic operations, cultures were incubated in BHIS containing sucrose and without antibiotics and spread onto BHIS agar plates to obtain bacteria losing two plasmids.

### Determination of genome editing efficiency

For convenient determination of the genome editing efficiency, two model strains *Cg*Del and *Cg*Int were constructed by the pk18*mobrpsL*-mediated method [47]. Deletion and integration of the designed DNA sequences at genomic levels of *Cg*Del and *Cg*Int, respectively, conferred kanamycin resistance. Therefore, transformants were spotted onto agar plates supplemented with kanamycin, and the editing efficiency was determined by calculating the fraction (%) of cells exhibiting kanamycin resistance. For the construction of D-PA producing strains, colony PCR was performed to determine the efficiencies of gene deletion/insertion.

### Fermentation and analysis for D-PA production

Strains were cultured on agar slants at 32 °C for more than 12 h. Seed cultures for shake-flask fermentation were prepared by transferring an appropriate number of agar-slant-cultured cells into a 500 mL Erlenmeyer flask containing 30 mL of medium and incubating at 32 °C with shaking at 220 rpm for 12–14 h. The seed cultures were transferred with 10% inoculum into a 500 mL baffled shake flask containing 30 mL of medium and incubated at 32 °C with shaking at 220 rpm for 48 h. The shake flask fermentation was conducted in the fed-batch mode. During the fermentation, pH was maintained at approximately 7.0 by adding NH_4_OH (25%, v/v) through a micro-injector; the phenol red included in the medium indicated the changes in pH. The feeding solution for the fed-batch culture was 60% (m/v) glucose and transfer pipettes were used for this assay.

The seed culture and the fed-batch fermentation media in the bioreactor were the same as those used for shake flask fermentation. Seed cultures were prepared by transferring an appropriate number of agar-slant-cultured cells into a 5-L fermenter (Baoxing, Shanghai, China) containing 3 L of seed medium. The pH was maintained constant at 7.0 through the automated addition of NH_4_OH (25%, v/v). Dissolved oxygen concentration was maintained at > 20% by varying the stirrer speed and aeration rate. The temperature was kept constant at 32 °C. The seed culture was continued until the OD_600_ of the culture reached nearly 14–16, and 500 mL of the culture broth was retained for the fed-batch fermentation. The pH and temperature in the fed-batch fermentation were controlled using the same procedures. When glucose was exhausted, 80% glucose solution was added at appropriate rates to keep the glucose concentration above 3 g/L, ensuring there is no lack of glucose. Samples were taken at a 2 h interval for determination of OD_600_ and the D-PA concentration.

Cell growth was monitored by measuring the OD_600_ of cultures [48]. To monitor the production of D-PA, samples were centrifuged at 13,000 rpm for 2 min, and the supernatant was diluted with deionized water and filtered with cellulose acetate membrane with 0.22 μm pores. The prepared samples were analyzed by high-performance liquid chromatography (HPLC) using an Agilent 1100 series as previously described [43] with a slight modification. The elution buffer was constituted of water, acetonitrile and phosphoric acid in a ratio of 947:50:3. The retention time for D-PA was 10.4 min.

### Statistical analysis

The data in this study represented mean values and standard deviations. The significant differences in data were determined via a one-way analysis of variance, followed by Student’s two-tailed *t*-*test*.

## Supplementary Information

**Additional file 1.** Supplementary methods and data.

## Data Availability

All data generated or analyzed during this study are included in this published article [and its additional files].

## Abbreviations

CRISPR: Clustered regularly interspaced short palindromic repeat
D-PA: D-pantothenic acid
DSB: Double-strand break
HR: Homologous recombination
PAM: Protospacer adjacent motif
RAPID: RecombinAtion Prior to Induced Double-strand-break
