# Supplementary material for: Enhanced production of d-pantothenic acid in Corynebacterium glutamicum using an efficient CRISPR–Cpf1 genome editing method
Source: Microb Cell Fact. 2023 Jan 6;22:3. doi: 10.1186/s12934-023-02017-1 (PMC9817396; doi:10.1186/s12934-023-02017-1)
Supplement: Supplementary file 1 — Additional file 1. Supplementary methods and data. [file 12934_2023_2017_MOESM1_ESM.docx]

**Supplementary Information for:**

**Enhanced production of D-pantothenic acid in *Corynebacterium glutamicum* using an efficient CRISPR–Cpf1 genome editing method**

Rui Su^a,1^, Ting Wang^a,1^, Taidong Bo^a^, Ningyun Cai^a^, Meng Yuan^a^, Chen Wu^a^, Hao Jiang^a^, Huadong Peng^c,^*, Ning Chen^a,b^, Yanjun Li^a,b,^*

^a^ College of Biotechnology, Tianjin University of Science and Technology, Tianjin 300457, China

^b^ Key Laboratory of Industrial Fermentation Microbiology, Ministry of Education, Tianjin University of Science and Technology, Tianjin 300457, China

^c^ The Novo Nordisk Foundation Center for Biosustainability, Technical University of Denmark, 2800 Kongens Lyngby, Denmark

* Corresponding author

1. mail address: yjli@tust.edu.cn (Y. J. Li); hdpeng@biosustain.dtu.dk (H.D.P).

^1^ These authors contributed equally to this work.

**Plasmid construction**

To generate pk18*mobrpsL*-kan*_del_, genomic DNA of *C. glutamicum* ATCC 13032 was used as template to amplify the upstream and downstream fragments of *cgl1890* using primers P1/P2 and P9/P10, respectively, pk18*mobrpsL* was used for amplifying two parts of kanamycin cassette using primers P3/P4 and P7/P8, genomic DNA of *E. coli* MG1655 was used for amplifying the 534 bp of partial *xylA* gene (*xylA**, Fig. S1a) using primers P5/P6, pk18*mobrpsL* was linearized using *Xba* I and *Kpn* I, and these five fragments were ligated by overlap-extension PCR, which was used for plasmid assembly by homologous recombination. To generate pk18*mobrpsL*-kan*_int_, upstream and downstream fragments of *cgl1890* were amplified using primers P1/P11 and P14/P4, respectively, the truncated kanamycin cassette without 193 bp C-terminal (Fig. S1b) was amplified using primers P12/P13 (the PAM and crRNA*_xylA_* were designed within primer P13), and these fragments were ligated and then assembled.

To generate pECspec, pEC-XK99E and pREDCas9^1^ were used as templates to amplify the backbone omitting the CDS sequence of *kan^R^* gene using primers P15/P16 (by reverse PCR), and the CDS sequence of *spec^R^* gene using primers P17/P18, respectively. These two fragments were purified and recovered for assembly by homologous recombination. To generate pEC-Redαβγ, pECspec was linearized using *Eco*R I and *Kpn* I, the P_hom_ sequence and *redαβγ* were amplified from *C. glutamicum* genomic DNA and pREDCas9 using primers P19/P20 and P21/P22, respectively, these two fragments were ligated and assembled with the linear plasmid. To generate pEC-RecET, the P_hom_ sequence was amplified using primers P19/P23 and *recET* was amplified from *E. coli* genomic DNA using primers P24/P25. To generate pEC-RecA, P_gapA_ and *recA* were amplified from the genomic DNA of *C. glutamicum* and *E. coli* using primers P26/P27 and P28/P29, respectively. To generate pEC-RecET-RecA, pEC-RecET was linearized using *Xba* I, pEC-RecA was used as a template to amplify P_gapA_-*recA* using primers P30/P31. The linear donor template for integration of a 528 bp sequence (donor_int_) was amplified from pXM-donor_int_ using primers P59/P60.

To generate Cpf1- and/or crRNA-expressing pXMJ19 derivatives, pXMJ19 was linearized by reverse PCR using primers P32/P33, omitting the sequences of *lacI* and P_tac_. *Fncpf1* gene^2^ was synthesized and assembled into pUC57 by GENEWIZ from Azenda Life Sciences. To generate pXM-plCpf1, *Fncpf1* was amplified using primers P34/P35 and P_lacM_ was designed in primer P34, and the P_lacM_-*Fncpf1* fragment was then assembled into linearized pXMJ19. To generate pXM-ptCpf1, pXtuf was linearized using *Hin*d III and *Bam*H I, *Fncpf1* was amplified using primers P36/P35. To generate pXM-plCpf1-crRNA, pXM-plCpf1 was linearized with *Bam*H I, and the P_j23119_-crRNA*_xylA_* oligonucleotides (O1) were synthesized as minigene by GENEWIZ and assembled into the linearized plasmid. Similarly, pXM-ptCpf1-crRNA was constructed using linearized pXM-ptCpf1. The crRNA was designed by the online tool CHOPCHOP, wherein the genome sequence of *C. glutamicum* ATCC 13032 was submitted by the authors’ group. To generate pXM-plCpf1-crRNA-donor_del_, pXM-plCpf1-crRNA was linearized using *Xba* I, left and right arms (Fig. S1a) were amplified from pk18*mobrpsL*-kan*_del_ using primers P37/P38 and P39/P40, respectively, ligated and then assembled with linearized plasmid. To generate pXM-plCpf1-crRNA-donor_int_, partial kan^R^ gene, partial *xylA* and *cgl1890* downstream fragment (Fig. S1b) were amplified from pK18*mobrpsL*, *E. coli* genomic DNA and pk18*mobrpsL*-kan*_del_ using primers P41/P42, P43/P44 and P45/P46, respectively, and these three fragments were ligated and assembled into linearized pXM-plCpf1-crRNA. Plasmids pXM-ptCpf1-crRNA-donor_del_ and pXM-ptCpf1-crRNA-donor_int_ were constructed similarly, using linearized pXM-ptCpf1-crRNA as backbone. Linear donor template for deletion of a 534 bp sequence (donor_del_) was amplified from pXM-plCpf1-crRNA-donor_del_ using primers P37/P40. Linear donor templates for integration of fragments of different lengths (approximately 1 kb, 2 kb, 3 kb, 4 kb, and 5 kb) were amplified from each corresponding pXM-donor derivative using primers P59/P60, as described for the preparation of donor_int_.

To generate pXM-ptCpf1-crRNA*_ldhA_*, a pair of reverse complementary single-stranded oligonucleotides (O2) were synthesized and annealed, and then assembled into linearized pXM-ptCpf1-crRNA. A linear donor template was generated by the ligation of upstream and downstream fragments of *ldhA*, which were amplified from *C. glutamicum* genomic DNA using primers P47/P48 and P49/P50, respectively. To generate pXM-ptCpf1-crRNA*_cg1890_*-*alaD*_int_, upstream and downstream fragments of *cg1890* and P_sod_ were amplified from *C. glutamicum* genomic DNA using primers P51/P52, P57/P58 and P53/P54, respectively, *alaD* was amplified from *B. subtilis* genomic DNA using primers P55/P56, and these fragments were ligated and then assembled into linearized pXM-ptCpf1-crRNA. The crRNA*_cg1890_* was designed in primer P51.

To generate pXM-donor_int_, partial *kan^R^* gene and *cgl1890* downstream fragment (Fig. S1b) were amplified from pk18*mobrpsL*-kan*_del_ using primers P59/P42 and P45/P60, respectively, donor sequence was amplified from *E. coli* genomic DNA using primers P43/P44, and these three fragments were ligated and assembled into the reverse PCR-linearized pXMJ19 plasmid. Other plasmids harboring donor templates of different lengths were constructed by a similar method, with varying reverse primers for donor fragments and forward primers for downstream fragments. For the integration of approximately 1 kb, 2 kb, 3 kb, 4 kb, and 5 kb fragments, primers P61 (reverse) and P62 (forward), P63 and P64, P65 and P66, P67 and P68, P69 and P70 were used, respectively.

To generate pXM-P_prp_Cpf1-crRNA, *prpR* and P_prpD2_ were amplified from *C. glutamicum* genomic DNA using primers P71/P72 and P73/P74, respectively, *Fncpf1*-P_j23119_-crRNA*_xylA_* was amplified from pXM-plCpf1-crRNA using P75/P76, and these three fragments were ligated and assembled into reverse-PCR-linearized pXMJ19. To generate pXM-P_BAD_Cpf1-crRNA, *araE* from *C. glutamicum* 31831 (GenBank: AB447371.1) was synthesized and inserted into pUC57 by GENEWIZ and was then amplified using primers P79/P80, P_hom_ was amplified from pEC-RecET using primers P77/P78, *araC* and P_BAD_ were amplified from *E. coli* genomic DNA using primers P81/P82, *Fncpf1*-P_j23119_-crRNA*_xylA_* was amplified from pXM-plCpf1-crRNA using primers P83/P76, and these three fragments were ligated and assembled into linearized pXMJ19. To generate pXM-P_tac_Cpf1-crRNA, pXMJ19 was linearized using *Hin*d III and *Bam*H I, *Fncpf1*-P_j23119_-crRNA*_xylA_* was amplified from pXM-plCpf1-crRNA using primers P84/P76, and the two fragments were assembled by homologous recombination.

To generate pEC-Cpf1-RecET, pEC-RecET was linearized using *Nco* I, *Fncpf1* was amplified from pXM-plCpf1 using primers P85/P86, and the two fragments were assembled by homologous recombination. To generate pXM-crRNA, oligonucleotides (O3) were synthesized as minigene and assembled into reverse-PCR linearized pXMJ19.

To generate pXMJ19ts, pXMJ19 was linearized using *Nde* I and *Hpa* I, two fragments were amplified with pXMJ19 as a template using primers P87/P88 and P89/P90, which were then ligated and assembled with the linearized plasmid. The C137T mutation was designed in primers P88 and P89. To generate pXM*sacB*-crRNA, pXM-crRNA was amplified by reverse PCR using primers P91/P92, *sacB* cassette was amplified with pk18*mobsacB* as a template using primers P93/94, and the two fragments were ligated by homologous recombination. To generate pECΔ*per1*-Cpf1-RecET, two fragments were amplified with pEC-Cpf1-RecET as template using primers P95/P96 and P97/P98 and ligated.

To generate pXM*sacB*-crRNA-donor_del_, pXM*sacB*-crRNA was linearized using *Xba* I, and the donor sequence was amplified with pXM-plCpf1-crRNA-donor_del_ as a template using primers P99/P40, and were assembled into linearized pXM*sacB*-crRNA.

To generate pECΔ*per1*-Cpf1-P_sod_-RecET, two fragments were amplified with pECΔ*per1*-Cpf1-RecET as template using primers P100/P101 and P104/P105, omitting P_hom_, and were ligated; P_sod_ was amplified from *C. glutamicum* genomic DNA using primers P102/P103, and was assembled into the ligated plasmid backbone. The pECΔ*per1*-Cpf1-P_gapA_-RecET was constructed similarly, except that P_gapA_ was amplified using primers P106/P107. To generate pECΔ*per1*-Cpf1-P_gapA_-theoE*-RecET, P_gapA_ was amplified using primers P106/P108, oligonucleotides containing theophylline riboswitch (O4) were synthesized and ligated with P_gapA_, which were then assembled into the plasmid backbone prepared as abovementioned.

To generate pXM*sacB*-crRNA-donor_int1kb_, donor sequence for integration of approximately 1 kb was amplified with pXM-donor_int1kb_ as a template using primers P109/P60, and were assembled into *Xba* I-linearized pXM*sacB*-crRNA. Other plasmids for the integration of DNA fragments of different lengths were constructed by a similar method. Donor sequences were amplified from each corresponding pXM-donor derivative using the same primer pair.

To generate pXM*sacB*-crRNA*_cg1995_*-*ilvBNC*_int_, pXM*sacB*-crRNA was linearized using *Xba* I, upstream and downstream fragments of *cg1995*, P_tuf_ and *ilvBNC* were amplified from the genomic DNA *C. glutamicum* XV (NCBI accession NZ_CP018175.1) using primers P110/P111, P116/P117, P112/P113 and P114/P115, respectively, and these fragments were ligated and then assembled into the linearized plasmid. The crRNA*_cg1995_* was designed in primer P110. To generate pXM*sacB*-crRNA*_ilvE_*, a pair of reverse complementary single-stranded oligonucleotides (O5) were synthesized and annealed, and then assembled into linearized pXM*sacB*-crRNA. The linear donor template for *ilvE* deletion was constructed by ligation of the upstream and downstream fragments of *ilvE*, which were amplified from *C. glutamicum* genomic DNA using primers P118/P119 and P120/P121. The pXM*sacB*-crRNA*_avtA_* was constructed in a similar way, in which oligonucleotides (O6) were involved. The upstream and downstream fragments of *avtA*, for linear donor template preparation, were amplified using primers P122/P123 and P124/P125. To generate pXM*sacB*-crRNA*_cg1960_*-*aspB*_int_, upstream and downstream fragments of *cg1960*, P_tuf_ and *aspB* were amplified from *C. glutamicum* genomic DNA using primers P126/P127, P132/P133, P128/P129 and P130/P131, respectively, and these fragments were ligated and then assembled into the linearized plasmid. To generate pXM*sacB*-crRNA*_Ncgl2850a_*-*aspC*_int_, upstream and downstream fragments of *Ncgl2850a* and P_tuf_ were amplified from *C. glutamicum* genomic DNA using primers P134/P135, P140/P142 and P136/P137, respectively, *apsA* was amplified from *E. coli* genomic DNA using primers P138/P139, and these fragments were ligated and then assembled. To generate pXM*sacB*-crRNA*_ilvA_*, oligonucleotides (O7) were synthesized and assembled into linearized pXM*sacB*-crRNA. A linear donor template for *ilvA* deletion was constructed by ligation of the corresponding upstream and downstream fragments amplified from *C. glutamicum* genomic DNA using primers P142/P143 and P144/P145. To generate pXtuf-*panBCD_Bsu_*, *panBCD* was amplified from the genomic DNA of *B. subtilis* 168 using primers P146/P147, and was assembled into *Hin*d III and *Bam*H I-linearized pXtuf.

**Strain construction**

To generate model strains *Cg*Del and *Cg*Int, plasmids pk18*mobrpsL*-kan*_del_ and pk18*mobrpsL*-kan*_int_ were introduced into competent cells of *C. glutamicum rpsL*^K43T^ (an *rpsL* mutant of the wild-type ATCC 13032 conferring streptomycin resistance), respectively, and single crossover strain and double crossover strain were selected by the successive application of kanamycin and streptomycin.^3^

To delete *ldhA* from the genome of *C. gltuamicum* ATCC 13032, pXM-ptCpf1-crRNA*_ldhA_* and the linear donor template were electroporated into competent cells harboring pEC-RecET, colonies were subjected to colony PCR and subsequent DNA sequencing to identify the correct *ldhA*-deleted strain (*Cg*Ala1). After curing of pXM-ptCpf1-crRNA*_ldhA_* by continuous passage without chloramphenicol supplementation, cells were made competent, and pXM-ptCpf1-crRNA*_cg1890_*-*alaD*_int_ was electroporated for integration of *alaD_Bsu_* (*Cg*Ala2). The two plasmids in *Cg*Ala2 were cured by a continuous passage in the absence of antibiotics.

**Alanine production**

For the production of alanine, the seed culture medium (in a 500-mL Erlenmeyer flask) contained (per liter) glucose 35 g, yeast extract (Oxoid) 5 g, corn steep liquor 20 mL, soybean protein hydrolysate 20 mL, KH_2_PO_4_ 2.0 g, MgSO_4_·7H_2_O 1.2 g, FeSO_4_·7H_2_O 20 mg, MnSO_4_·H_2_O 20 mg, VB_1_ 0.5 mg, V_H_ 0.1 mg (pH adjusted to 7.0-7.2). Alanine shake flask fermentation medium contained (per liter) glucose 60 g, corn steep liquor 30 mL, soybean protein hydrolysate 30 mL, KH_2_PO_4_ 2.5 g, MgSO_4_·7H_2_O 1.6 g, FeSO_4_·7H_2_O 20 mg, MnSO_4_·H_2_O 20 mg, VB_1_ 0.5 mg, V_H_ 0.05 mg (pH 7.0-7.2).

Shake flask fermentation was performed as described for D-PA production. The alanine in cultures was quantified through HPLC analyses performed using precolumn derivatization, as previously described.^4^

**References**

1. Li, Y. F., Lin, Z. Q., Huang, C., Zhang, Y., Wang, Z. W., Tang, Y. J., Chen, T., Zhao, X. M. Metabolic engineering of *Escherichia coli* using CRISPR-Cas9 meditated genome editing. *Metab. Eng.* **2015**, 31, 13–21.
2. Jiang, Y., Qian, F. H., Yang, J. J., Liu, Y. M., Dong, F., Xu, C. M., Sun, B. B., Chen, B., Xu, X. S., Li, Y., Wang, R. X., Yang, S. CRISPR-Cpf1 assisted genome editing of *Corynebacterium glutamicum*. *Nat. Commun.* **2017**, 8, 15179.
3. Wang, T., Li, Y. J., Li J., Zhang D. Z., Cai N. Y., Zhao G. H., Ma H. K., Shang C., Ma Q., Xu, Q. Y., Chen, N. An update of the suicide plasmid-mediated genome editing system in *Corynebacterium glutamicum*. *Microb. Biotechnol.* **2019**, 12, 907–919.
4. Zhang, C. L., Li, Y. J., Ma, J., Liu, Y., He, J. L., Li, Y. Z., Zhu, F. Z., Meng, J., Zhan, J. J., Li, Z. X., Zhao, L., Ma, Q., Fan, X. G., Xu, Q. Y., Xie, X. X., Chen, N. High production of 4-hydroxyisoleucine in *Corynebacterium glutamicum* by multistep metabolic engineering. *Metab. Eng.* **2018**, 49, 287–298.

**Table S1** **Strains and plasmids used in this study**

| **Name** | **Genotype or characteristic** | **Source** |
| --- | --- | --- |
| **Strains** |  |  |
| *E. coli* DH5α | F^-^, △(lacZYA-*argF*) U169, *hsdR17* (rk^-^mk^+^), *recA1*, *endA1*, *relA1* | Lab stock |
| *E. coli* MG1655 | wild-type (WT) *E. coli* strain | Lab stock |
| *B. subtilis* 168 | WT *B. subtilis* strain | Lab stock |
| *C. glutamicum* ATCC 13032 | WT *C. glutamicum* strain | Lab stock |
| *C. glutamicum rpsL*^K43T^ | *rpsL*^K43T^ mutant of WT, confers streptomycin resistance | [3] |
| *Cg*Del | deletion model strain: *rpsL*^K43T^*cgl1890*::inactivated kanamycin cassette (by 534 bp *xylA_Eco_** insertion), for fast identification of gene deletion by kanamycin selection | This study |
| *Cg*Int | integration model strain: *rpsL*^K43T^*cgl1890*::truncated kanamycin cassette (lack of C-terminal 193 bp), for fast identification of gene integration by kanamycin selection | This study |
| *Cg*Ala1 | WTΔ*ldhA* | This study |
| *Cg*Ala2 | *Cg*Ala1*cgl1890*::P_sod_-*AlaD_Bsu_* | This study |
| Pan-1 | WT*cg1995*::P_tuf_-XV*ilvBNC*Δ*ilvE*Δ*avtA* | This study |
| Pan-2 | Pan-1*cg1960*::*aspB* | This study |
| Pan-3 | Pan-2*Ncg2850a*::*aspA_Eco_* | This study |
| Pan-4 | Pan-3*cg1960*::Δ*ilvA* | This study |
| **Plasmids** |  |  |
| pK18*mobsacB* | suicide vector containing *B. subtilis* *sacB* gene, kan^R^ | Lab stock |
| pK18*mobrpsL* | derived from suicide vector pK18*mobsacB*, *sacB* was substituted by P_tuf_-*rpsL* cassette, kan^R^ | [3] |
| pXMJ19 | *E. coli*/*C. glutamicum* shuttle cloning vector, cm^R^ | Lab stock |
| pEC-XK99E | *E. coli*/*C. glutamicum* shuttle cloning vector, kan^R^ | Lab stock |
| pREDCas9 | Cas9 and λ Red expression vector, Spe^R^ | [1] |
| pk18*mobrpsL*-kan*_del_ | for construction of *Cg*Del | This study |
| pk18*mobrpsL*-kan*_int_ | for construction of *Cg*Int | This study |
| pECspec | pEC-XK99E with kanamycin-expressing cassette substituted by spectinomycin cassette | This study |
| pEC-Redαβγ | pECspec-P_hom_-*redαβγ* | This study |
| pEC-RecET | pECspec-P_hom_-*recET* | This study |
| pEC-RecA | pECspec-P_gapA_-*recA* | This study |
| pEC-RecET-RecA | pECspec-P_hom_-*recET*-P_gapA_-*recA* | This study |
| pXtuf | derived from pXMJ19, *lacI* and P_tac_ replaced by P_tuf_, Cm^R^ | Lab stock |
| pXM-plCpf1 | pXM-P_lacM_-*Fncpf1* | This study |
| pXM-plCpf1-crRNA | pXM-P_lacM_-*Fncpf1*-P_j23119_-crRNA*_xylA_* | This study |
| pXM-plCpf1-crRNA-donor_del_ | pXM-P_lacM_-*Fncpf1*-P_j23119_-crRNA*_xylA_*-UH-DH_del534bp_ | This study |
| pXM-plCpf1-crRNA-donor_int_ | pXM-P_lacM_-*Fncpf1*-P_j23119_-crRNA*_xylA_*-UH-528bp-DH | This study |
| pXM-ptCpf1 | pXM-P_tuf_-*Fncpf1* | This study |
| pXM-ptCpf1-crRNA | pXM-P_tuf_-*Fncpf1*-P_j23119_-crRNA*_xylA_* | This study |
| pXM-ptCpf1-crRNA-donor_del_ | pXM-P_tuf_-*Fncpf1*-P_j23119_-crRNA*_xylA_*-UH-DH_del534bp_ | This study |
| pXM-ptXpf1-crRNA-donor_int_ | pXM-P_tuf_-*Fncpf1*-P_j23119_-crRNA*_xylA_*-UH-528bp-DH | This study |
| pXM-ptCpf1-crRNA*_ldhA_* | pXM-P_tuf_-*Fncpf1*-P_j23119_-crRNA*_ldhA_* | This study |
| pXM-ptCpf1-crRNA*_cg1890_*-*alaD*_int_ | pXM-P_tuf_-*Fncpf1*-P_j23119_-crRNA*_cg1890_*-UH-P_sod_-*alaD_Bsu_*-DH | This study |
| pXM-donor_int_ | pXM-UH-int528bp-DH | This study |
| pXM-donor_int1kb_ | pXM-UH-int1015bp-DH | This study |
| pXM-donor_int2kb_ | pXM-UH-int2147bp-DH | This study |
| pXM-donor_int4kb_ | pXM-UH-int4115bp-DH | This study |
| pXM-P_prp_Cpf1-crRNA | pXM-*prpR-*P_prpD2-_*Fncpf1*-P_j23119_-crRNA*_xylA_* | This study |
| pXM-P_BAD_Cpf1-crRNA | pXM-P_hom_-*araE*-*araC-*P_BAD-_*Fncpf1*-P_j23119_-crRNA*_xylA_* | This study |
| pXM-P_tac_Cpf1-crRNA | pXMJ19-*Fncpf1*-P_j23119_-crRNA*_xylA_* | This study |
| pEC-Cpf1-RecET | pECspec-*Fncpf1*-P_hom_-*recET*, *Fncpf1* expressed under the IPTG inducible P_trc_ promoter | This study |
| pXM-crRNA | pXM-P_j23119_-crRNA*_xylA_* | This study |
| pXMJ19ts | temperature-sensitive pXMJ19 | This study |
| pXM*sacB*-crRNA | pXM-*sacB*-P_j23119_-crRNA | This study |
| pECΔ*per1*-Cpf1-RecET | pECspecΔ*per1*-*Fncpf1*-P_hom_-*recET* | This study |
| pXM*sacB*-crRNA-donor_del_ | pXM-*sacB*-P_j23119_-crRNA*_xylA_*-UH-DH_del534bp_ | This study |
| pECΔ*per1*-Cpf1-P_sod_-RecET | pECspecΔ*per1*-P_sod_-*Fncpf1*-P_sod_-*recET* | This study |
| pECΔ*per1*-Cpf1-P_gapA_-RecET | pECspecΔ*per1*-P_gapA_-*Fncpf1*-P_sod_-*recET* | This study |
| pECΔ*per1*-Cpf1-P_gapA_-theoE*-RecET | pECspecΔ*per1*-*Fncpf1*-P_gapA_-theoE*-*recET*, recET expressed under the control of theophylline riboswitch | This study |
| pXM*sacB*-crRNA-donor_int1kb_ | pXM*sacB*-P_j23119_-crRNA*_xylA_*-UH-1015bp-DH | This study |
| pXM*sacB*-crRNA-donor_int2kb_ | pXM*sacB*-P_j23119_-crRNA*_xylA_*-UH-2147bp-DH | This study |
| pXM*sacB*-crRNA-donor_int3kb_ | pXM*sacB*-P_j23119_-crRNA*_xylA_*-UH-3212bp-DH | This study |
| pXM*sacB*-crRNA-donor_int4kb_ | pXM*sacB*-P_j23119_-crRNA*_xylA_*-UH-4115bp-DH | This study |
| pXM*sacB*-crRNA-donor_int5kb_ | pXM*sacB*-P_j23119_-crRNA*_xylA_*-UH-5156bp-DH | This study |
| pXM*sacB*-crRNA*_cg1995_*-*ilvBNC*_int_ | pXM*sacB*-P_j23119_-crRNA*_xylA_*-UH-P_tuf_-XV*ilvBNC*-DH | This study |
| pXM*sacB*-crRNA*_ilvE_* |  | This study |
| pXM*sacB*-crRNA*_avtA_* |  | This study |
| pXM*sacB*-crRNA*_cg1960_*-*aspB*_int_ | pXM*sacB*-crRNA*_cg1960_*-UH-P_tuf_-*aspB*-DH | This study |
| pXM*sacB*-crRNA*_Ncgl2850a_*-*aspC*_int_ | pXM*sacB*-crRNA*_Ncgl2850a_*-UH-P_tuf_-*aspC_Eco_*-DH | This study |
| pXM*sacB*-crRNA*_ilvA_* |  | This study |
| pXtuf-*panBCD_Bsu_* | *B. subtilis panBCD* overexpressing plasmid, cm^R^ | This study |

**Table S2** **Primers and oligonucleotides used in this study**

| **Primers and oligonucleotides** | **Sequence** (5’-3’) |
| --- | --- |
| P1 | tgcctgcaggtcgacTCTAGAGACTAGTGGGGGTTTCTGCTGTT |
| P2 | gttccgcttcctttagcagcCCTGGCGATAGGTGTCAAGAATTCG |
| P3 | cgaattcttgacacctatcgccaggGCTGCTAAAGGAAGCGGAAC |
| P4 | gctggtcaaaataggcttgcaCATGATATTCGGCAAGCAGGCAT |
| P5 | atgcctgcttgccgaatatcatgTGCAAGCCTATTTTGACCAGC |
| P6 | gaaaagcggccattttccacTATGGGTTGCTTCCATCGCT |
| P7 | agcgatggaagcaacccataGTGGAAAATGGCCGCTTTTC |
| P8 | atagcgtggttggcgaagttCGCGATTTACTTTTCGACCTC |
| P9 | gaggtcgaaaagtaaatcgcgAACTTCGCCAACCACGCTAT |
| P10 | tacgaattcgagcgcGGTACCAATGGTAGTGAACTACCGTCCCTT |
| P11 | ctttctacgtgttccgcttcctCCTGGCGATAGGTGTCAAGAATTCG |
| P12 | cgaattcttgacacctatcgccaggAGGAAGCGGAACACGTAGAAAG |
| P13 | atagcgtggttggcgaagtt*TTTA*CGCTTCGCCAACGCCAGTGCCTCACCATTTTCCACCATGATATTCG (*PAM*+crRNA*_xylA_*) |
| P14 | ctggcgttggcgaagcgtaaaAACTTCGCCAACCACGCTAT |
| P15 | GCGAAACGATCCTCATCCTG |
| P16 | GCGGGACTCTGGGGTTCG |
| P17 | caggatgaggatcgtttcgcATGCGCTCACGCAACTGG |
| P18 | cgaaccccagagtcccgcTTATTTGCCGACTACCTTGGTGA |
| P19 | aggaaacagaccatgGAATTCTGTAAGGCCTGCACCAACAAT |
| P20 | atctcagtttcagtattaatatccatGATTCTCCAAAAATAATCGCGGT |
| P21 | accgcgattatttttggagaatcATGGATATTAATACTGAAACTGAGATCAAG |
| P22 | tctagaggatccccgGGTACCTCATCGCCATTGCTCCCC |
| P23 | AAGAGTGGTTTTGTGCTCATGATTCTCCAAAAATAATCGCGG |
| P24 | CCGCGATTATTTTTGGAGAATCATGAGCACAAAACCACTCTT |
| P25 | tagaggatccccgGGTACCTTATTCCTCTGAATTATCGATTACACTG |
| P26 | ggcaaatattctgaaatgagctgGAATTCATGATTGAAGCCTAAAAACGACC |
| P27 | gctgtcttcttgggagccatGTTGTGTCTCCTCTAAAGATTGTAGG |
| P28 | cctacaatctttagaggagacacaacATGGCTCCCAAGAAGACAGC |
| P29 | tctagaggatccccgGGTACCTTAGTCTTCAGCGTCTGCTTCG |
| P30 | atcgataattcagaggaataaTCTAGAATGATTGAAGCCTAAAAACGACC |
| P31 | tgcctgcaggtcgacTCTAGATTAGTCTTCAGCGTCTGCTTCG |
| P32 | CTCACTGGTGAAAAGAAAAACCAC |
| P33 | CTAGAGGATCCCCGGGTACCGAGCTC |
| P34 | gtggtttttcttttcaccagtgagATCGTGTGGTACCATGTGTGGAATTGGAAAGGACTTGAACGATGTCCATCTACCAAGAGTTTGTGAA (P_lacM_) |
| P35 | gagctcggtacccggggatcctctagTTATTGCGGTTCTGGACAAAT |
| P36 | cgaagtccaggaggaaagcttATGTCCATCTACCAAGAGTTTGTGA |
| O1 | tgtccagaaccgcaataactaaTTGACAGCTAGCTCAGTCCTAGGTATAATCCCGGGG*AATTTCTACTGTTGTAGAT*CGCTTCGCCAACGCCAGTGCCTCATCTAGAggctgttttggcggatga (P_j23119_+*direct repeat*+crRNA*_xylA_*+*Xba* I) |
| P37 | ccaacgccagtgcctcatctagaTTGCACGCAGGTTCTCCG |
| P38 | GAAAAGCGGCCATTTTCCACCATGATATTCGGCAAGCAGG |
| P39 | CCTGCTTGCCGAATATCATGGTGGAAAATGGCCGCTTTTC |
| P40 | ctctcatccgccaaaacagccCGCGATTTACTTTTCGACCTC |
| P41 | aacgccagtgcctcatctagaACAACAGACAATCGGCTGCTC |
| P42 | GGAAGCAATAAAATGGCACATGATCCTCTAGCGAACCCCAGAG |
| P43 | CTCTGGGGTTCGCTAGAGGATCATGTGCCATTTTATTGCTTCC |
| P44 | ATAGCGTGGTTGGCGAAGTTGTTTCGTAACCTTCACGACCG |
| P45 | CGGTCGTGAAGGTTACGAAACAACTTCGCCAACCACGCTAT |
| P46 | caaaacagccaagctgaattcAATGGTAGTGAACTACCGTCCCTT |
| O2 | gg*aatttctactgttgtagat*CGAAGACACCCGCGACGCTGCCTAtctagaggctgttttggcggatga (*direct repeat*+crRNA*_ldhA_*) |
| P47 | GGTGAGCAGAGACGCTAGTCTG |
| P48 | CGTCTTGGTTCTGCAGGATTGCGCCGGCACAAATGACA |
| P49 | GTCATTTGTGCCGGCGCAATCCTGCAGAACCAAGACG |
| P50 | GAAGATGCGCGTAATGCATG |
| P51 | g*aatttctactgttgtagat*GGTTTTCCTGAAGCAGATGAAACATCTAGATTCAGATGTTATCGAGTTACCGGA (*direct repeat*+crRNA*_cg1890_*) |
| P52 | ATTTTCGAAAGGAACATTCCTGTTGAGCCGCCAAGTTCTTCGTA |
| P53 | TACGAAGAACTTGGCGGCTCAACAGGAATGTTCCTTTCGAAAAT |
| P54 | AGCACCGGTCTCGATGTACATGGGTAAAAAATCCTTTCGTAGGTT |
| P55 | AACCTACGAAAGGATTTTTTACCCATGTACATCGAGACCGGTGCT |
| P56 | ATTGTGCACGCTGGGAAAACTTATTGAATCAGCTCGTCGACG |
| P57 | CGTCGACGAGCTGATTCAATAAGTTTTCCCAGCGTGCACAAT |
| P58 | ctctcatccgccaaaacagccCGTGTTCGATTCCACCAGGT |
| P59 | gtggtttttcttttcaccagtgagACAACAGACAATCGGCTGCTC |
| P60 | gagctcggtacccggggatcctctagAATGGTAGTGAACTACCGTCCCTT |
| P61 | ATAGCGTGGTTGGCGAAGTTGCCGATATGACCGTAAAACAGAT |
| P62 | ATCTGTTTTACGGTCATATCGGCAACTTCGCCAACCACGCTAT |
| P63 | ATAGCGTGGTTGGCGAAGTTTCTGGCTTGCTTAAGAACCCTT |
| P64 | AAGGGTTCTTAAGCAAGCCAGAAACTTCGCCAACCACGCTAT |
| P65 | ATAGCGTGGTTGGCGAAGTTGCGTTTAAAACACCGATAAACAA |
| P66 | CGGCCCACAGTAAAGATACCAAACTTCGCCAACCACGCTAT |
| P67 | ATAGCGTGGTTGGCGAAGTTTGGTATCTTTACTGTGGGCCG |
| P68 | TTGTTTATCGGTGTTTTAAACGCAACTTCGCCAACCACGCTAT |
| P69 | ATAGCGTGGTTGGCGAAGTTGGTGTTATAACGTTTTGCCGC |
| P70 | GCGGCAAAACGTTATAACACCAACTTCGCCAACCACGCTAT |
| P71 | gtggtttttcttttcaccagtgagCAATGGAGTGGATGACTGCTTCG |
| P72 | AGTCTTTTTTGGGGTGGCCTTTTAGTTGAGGAGTTGGACAGGG |
| P73 | CCCTGTCCAACTCCTCAACTAAAAGGCCACCCCAAAAAAGACT |
| P74 | CAAACTCTTGGTAGATGGACATGGCTGGAATGTGACTCCTTCTC |
| P75 | GAGAAGGAGTCACATTCCAGCCATGTCCATCTACCAAGAGTTTG |
| P76 | gagctcggtacccggggatcctctagTGAGGCACTGGCGTTGGCGAAGCG |
| P77 | gtggtttttcttttcaccagtgagTGTAAGGCCTGCACCAACAAT |
| P78 | GCAGATTCCGTATTGATAGTAACCATGATTCTCCAAAAATAATCGCGGT |
| P79 | ACCGCGATTATTTTTGGAGAATCATGGTTACTATCAATACGGAATCTGC |
| P80 | TGATGTAGCCGTCAAGTTGTCATAATCAGACGCCGATATTTCTCAAC |
| P81 | GTTGAGAAATATCGGCGTCTGATTATGACAACTTGACGGCTACATCA |
| P82 | CAAACTCTTGGTAGATGGACATATGGAGAAACAGTAGAGAGTTGCGA |
| P83 | TCGCAACTCTCTACTGTTTCTCCATATGTCCATCTACCAAGAGTTTG |
| P84 | ttctgaaatgagctgaagcttATGTCCATCTACCAAGAGTTTG |
| P85 | acacaggaaacagaccatggATGTCCATCTACCAAGAGTTTG |
| P86 | gtgcaggccttacagaattcTTAGTTATTGCGGTTCTGGACA |
| O3 | gtggtttttcttttcaccagtgagTTGACAGCTAGCTCAGTCCTAGGTATAATCCCGGGG*AATTTCTACTGTTGTAGAT*CGCTTCGCCAACGCCAGTGCCTCAtctagaggatccccgggtaccgagct (P_j23119_+*direct repeat*+crRNA*_xylA_*+*Xba* I) |
| P87 | gcgagctaccaactcatatgCACGGGGGCCACATAACC |
| P88 | CATGACTTCCAATTCAGCCAGAGGTAACCCCCAGCGAGAGTGAGAGT |
| P89 | ACTCTCACTCTCGCTGGGGGTTACCTCTGGCTGAATTGGAAGTCATG (C137T) |
| P90 | tgttatatcccgccgttaaCACCATCAAACAGGATTTTCGC |
| P91 | AACGTAAATGCCGCTTCGCC |
| P92 | AGGGCAATCAGCTGTTGCCC |
| P93 | gggcaacagctgattgccctTTATTTGTTAACTGTTAATTGTCCTTGTTC |
| P94 | ggcgaagcggcatttacgttCACATATACCTGCCGTTCACTATTATT |
| P95 | TCGCTCAAGGCGCACTCCCGTTCTGGATAATGTTTTTTGCG |
| P96 | TGGCCTGAAGCATCAGCGGTGGAGGATCGCATCAGCTG |
| P97 | CAGCTGATGCGATCCTCCACCGCTGATGCTTCAGGCCA |
| P98 | CGCAAAAAACATTATCCAGAACGGGAGTGCGCCTTGAGCGA |
| P99 | cgcttcgccaacgccagtgcctcaTTGCACGCAGGTTCTCCG |
| P100 | TCGCCCTAAAACAAAGTTAAACATCATGAGGGAAGCGGTGATCG |
| P101 | GGTACCTTAGTTATTGCGGTTCTGG |
| P102 | accgcaataactaaggtaccAACAGGAATGTTCCTTTCGAAAA |
| P103 | aagagtggttttgtgctcatGGGTAAAAAATCCTTTCGTAGGTTT |
| P104 | ATGAGCACAAAACCACTCTTCCTG |
| P105 | CGATCACCGCTTCCCTCATGATGTTTAACTTTGTTTTAGGGCGA |
| P106 | accgcaataactaaggtaccGAAGCCAGTGTGAGTTGCATCA |
| P107 | aagagtggttttgtgctcatGTTGTGTCTCCTCTAAAGATTGTAGGA |
| P108 | aagacgatgctggtatcaccTGTCCTCAACTTTAGCGGGGA |
| O4 | *ggtgataccagcatcgtcttGATGCCCTTGGCAGCACCCTGCTAAGGAGGCAACAAG*atgagcacaaaaccactcttcctg (*theophylline riboswitch theoE**) |
| P109 | cgcttcgccaacgccagtgcctcaACAACAGACAATCGGCTGCTC |
| P110 | gg*aatttctactgttgtagat*GTGCGATTGCTCGCGAGGGTGAGGCTTCTTGCTAGTTGTCAGTGGTCAA (*direct repeat*+crRNA*_cg1995_*) |
| P111 | CCTTCGGATCTAAACGATCTGTTAACTTGCGAGATGCTCATCAACAA |
| P112 | TTGTTGATGAGCATCTCGCAAGTTAACAGATCGTTTAGATCCGAAGG |
| P113 | TGTTGAGAAGCTGCCACATTCACAAGCTTTCCTCCTGGACTTCG |
| P114 | CGAAGTCCAGGAGGAAAGCTTGTGAATGTGGCAGCTTCTCAACA |
| P115 | CAACACTCTCTTCGGACAGTGATAGTTAAGCGGTTTCTGCGCG |
| P116 | CGCGCAGAAACCGCTTAACTATCACTGTCCGAAGAGAGTGTTG |
| P117 | tccgccaaaacagcctctagaGAGTAAGGGCGATATTGTTACCAAG |
| O5 | gg*aatttctactgttgtagat*CGTCCTGATGAAAACGCCGAGCGtctagaggctgttttggcgga (*direct repeat*+crRNA*_ilvE_*) |
| P118 | CGGCGAGTTCGATGGAAT |
| P119 | CCATTTCTTCGATGTACTTGTGCTGCAGGATCCATAGGAATCGG |
| P120 | CCGATTCCTATGGATCCTGCAGCACAAGTACATCGAAGAAATGG |
| P121 | CTTCCACCCCCTACGTCTCATA |
| O6 | gg*aatttctactgttgtagat*CTGGGCTGGTGACAATAACAGCCTtctagaggctgttttggcgga (*direct repeat*+crRNA*_avtA_*) |
| P122 | CAGCCATGACCAGGCTTTGGTACTG |
| P123 | GTTTATCGCCACCTTGGATCACAAGGAAGACACCATTGAAGGTGTGCG |
| P124 | CGCACACCTTCAATGGTGTCTTCCTTGTGATCCAAGGTGGCGATAAAC |
| P125 | CAGATAACGTTCTTCACGCTGGTAC |
| P126 | *ggaatttctactgttgtagat*GATCTGCGGGCAGGTCTACTGAGCcgcagatgtagccctccacaat (*direct repeat*+crRNA*_cg1960_*) |
| P127 | CCTTCGGATCTAAACGATCTGTTAACGTCTGAGACGTTGTAGGCAATGAGA |
| P128 | TCTCATTGCCTACAACGTCTCAGACGTTAACAGATCGTTTAGATCCGAAGG |
| P129 | CCGATCATCACAATGTTCAGCATAAGCTTTCCTCCTGGACTTCG |
| P130 | CGAAGTCCAGGAGGAAAGCTTATGCTGAACATTGTGATGATCGG |
| P131 | GGTCGGTGATGTTGTCATAGAAGAATTAGATTGAAATGGCATGGGC |
| P132 | GCCCATGCCATTTCAATCTAATTCTTCTATGACAACATCACCGACC |
| P133 | tccgccaaaacagcctctagaGCCACTGTGTGTAGATCTTGATCAT |
| P134 | *ggaatttctactgttgtagat*GAAAGCTATCTCGCGACGGTGGGGaacacctacacggacaaggacatct (*direct repeat*+crRNA*_Ncgl2850a_*) |
| P135 | CAGTGCAGACGAAAAGGTATTGCCCCTTCTTAGGGTTACTTTCGACTGCT |
| P136 | GGGCAATACCTTTTCGTCTGCACTGAGTGGGGTAGCGGCTTGTTAGA |
| P137 | CGCTTTTGAAGCAGAAGCCTACCTTTGTATGTCCTCCTGGACTTCGTG |
| P138 | AAGGTAGGCTTCTGCTTCAAAAGCGATGTCAAACAACATTCGTATCGAAG |
| P139 | GGCCATACAGCTGGTTCGCTTCTTATTACTGTTCGCTTTCATCAGTATAGCG |
| P140 | TAAGAAGCGAACCAGCTGTATGGCCCTTCAACAGCAAGCTGATCACTATG |
| P141 | tccgccaaaacagcctctagaGAGCGGATTAGCCATTATCAGTCAC |
| O7 | *ggaatttctactgttgtagat*TCTCCTTGGTGGTCACTGGCAATAtctagaggctgttttggcgga (*direct repeat*+crRNA*_ilvA_*) |
| P142 | TGGCAATAAATATGCGGATTTACTA |
| P143 | AAGTTCACCAAGAAGTAGTGCTTCATGGAGTCTGCACAGGAACATAGATG |
| P144 | CATCTATGTTCCTGTGCAGACTCCATGAAGCACTACTTCTTGGTGAACTT |
| P145 | GCTTACAAGCAGCTGTTGCACCTGC |
| P146 | cgaagtccaggaggaaagcttATGAAAACAAAACTGGATTTTCTAAAA |
| P147 | gagctcggtacccggggatccCTACAAAATTGTACGGGCTGGTT |

Lowercase letters indicate the overlapping sequences designed for overlap extension PCR or plasmid ligation by homologous recombination.

**(a)**


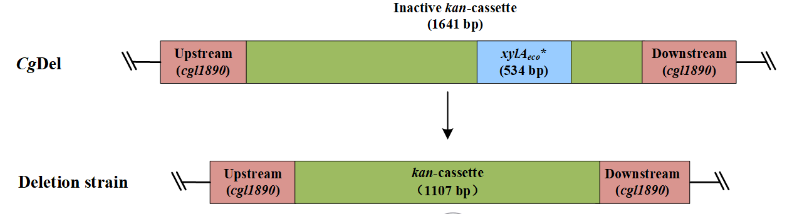


***Cg*Del:**

GACTAGTGGGGGTTTCTGCTGTTATGTTGCTGGCCATCGGGGTTGCATCTCCGGTGGCTCAAGCACAGGTGGAAGATCAATTTGAGCTTGTAGAAGAAATCACTGATGAGTAGTTTGCTGATGACGGTGTTGATTATGTTCCCAATAGGAATGCTCCAACAGTTGAGGAACAACTTGAGGATTACGAAACAGCACATCCAGAAGTAGTCGTTGAGTATCACGAGCAAGTCAACGATAGTAAAGATAATGTCGAGGAACTCCCGCTGCCTAAGCGGGACATAGTTGCAGGGGACATGCGTTCAGATGTTATCGAGTTACCGGAGGGGGTAAGCAAGGAGAAAGCTGACCAGCTAGAAGTTGCGGAAGCGCGACTTAACGAGGGTGCACGACTGATGGCAACCACCGGGTGTGAGGTTATGTGGCCAACGGGCTTCTCAGTTTGTGGCCGAATTCTTGACACCTATCGCCAGGGCTGCTAAAGGAAGCGGAACACGTAGAAAGCCAGTCCGCAGAAACGGTGCTGACCCCGGATGAATGTCAGCTACTGGGCTATCTGGACAAGGGAAAACGCAAGCGCAAAGAGAAAGCAGGTAGCTTGCAGTGGGCTTACATGGCGATAGCTAGACTGGGCGGTTTTATGGACAGCAAGCGAACCGGAATTGCCAGCTGGGGCGCCCTCTGGTAAGGTTGGGAAGCCCTGCAAAGTAAACTGGATGGCTTTCTTGCCGCCAAGGATCTGATGGCGCAGGGGATCAAGATCTGATCAAGAGACAGGATGAGGATCGTTTCGCATGATTGAACAAGATGGATTGCACGCAGGTTCTCCGGCCGCTTGGGTGGAGAGGCTATTCGGCTATGACTGGGCACAACAGACAATCGGCTGCTCTGATGCCGCCGTGTTCCGGCTGTCAGCGCAGGGGCGCCCGGTTCTTTTTGTCAAGACCGACCTGTCCGGTGCCCTGAATGAACTCCAAGACGAGGCAGCGCGGCTATCGTGGCTGGCCACGACGGGCGTTCCTTGCGCAGCTGTGCTCGACGTTGTCACTGAAGCGGGAAGGGACTGGCTGCTATTGGGCGAAGTGCCGGGGCAGGATCTCCTGTCATCTCACCTTGCTCCTGCCGAGAAAGTATCCATCATGGCTGATGCAATGCGGCGGCTGCATACGCTTGATCCGGCTACCTGCCCATTCGACCACCAAGCGAAACATCGCATCGAGCGAGCACGTACTCGGATGGAAGCCGGTCTTGTCGATCAGGATGATCTGGACGAAGAGCATCAGGGGCTCGCGCCAGCCGAACTGTTCGCCAGGCTCAAGGCGCGGATGCCCGACGGCGAGGATCTCGTCGTGACCCATGGCGATGCCTGCTTGCCGAATATCATGTGCAAGCCTATTTTGACCAGCTCGATCGCGTTCGTTATGAAGGCTCAAAATCCTCAAACCCGTTAGCATTCCGTCACTACAATCCCGACGAACTGGTGTTGGGTAAGCGTATGGAAGAGCACTTGCGTTTTGCCGCCTGCTACTGGCACACCTTCTGCTGGAACGGGGCGGATATGTTTGGTGTGGGGGCGTTTAATCGTCCGTGGCAGCAGCCTGGTGAGGCACTGGCGTTGGCGAAGCGTAAAGCAGATGTCGCATTTGAGTTTTTCCACAAGTTACATGTGCCATTTTATTGCTTCCACGATGTGGATGTTTCCCCTGAGGGCGCGTCGTTAAAAGAGTACATCAATAATTTTGCGCAAATGGTTGATGTCCTGGCAGGCAAGCAAGAAGAGAGCGGCGTGAAGCTGCTGTGGGGAACGGCCAACTGCTTTACAAACCCTCGCTACGGCGCGGGTGCGGCGACGAACCCAGATCCTGAAGTCTTCAGCTGGGCGGCAACGCAAGTTGTTACAGCGATGGAAGCAACCCATAGTGGAAAATGGCCGCTTTTCTGGATTCATCGACTGTGGCCGGCTGGGTGTGGCGGACCGCTATCAGGACATAGCGTTGGCTACCCGTGATATTGCTGAAGAGCTTGGCGGCGAATGGGCTGACCGCTTCCTCGTGCTTTACGGTATCGCCGCTCCCGATTCGCAGCGCATCGCCTTCTATCGCCTTCTTGACGAGTTCTTCTGAGCGGGACTCTGGGGTTCGCTAGAGGATCGATCCTTTTTAACCCATCACATATACCTGCCGTTCACTATTATTTAGTGAAATGAGATATTATGATATTTTCTGAATTGTGATTAAAAAGGCAACTTTATGCCCATGCAACAGAAACTATAAAAAATACAGAGAATGAAAAGAAACAGATAGATTTTTTAGTTCTTTAGGCCCGTAGTCTGCAAATCCTTTTATGATTTTCTATCAAACAAAAGAGGAAAATAGACCAGTTGCAATCCAAACGAGAGTCTAATAGAATGAGGTCGAAAAGTAAATCGCGAACTTCGCCAACCACGCTATGGCTGAGGCCGTAGTAGATAAAGCGGTGATTGATTATGGCTCATCGCCAGGAACCAGCTATTACAAGTTCGAGAAAACGGTGTACTTTCTAGATTGCAGAACTTATACATTCAATAAGAACTCAGGATGTAAAGAAATGCACGCTCCGCAATGGGTGACTATTATTTACAATCCTCATACTTTCACTGGAGCAAATTCGAACAGACCCAAGGGGGTAATTTCAGCATGGTGTAATTCAACCCCACCTGGTGGAATCGAACACGAGCCGGAAATTTCCCAATGTCCTGATCATGTGAATCTTTATAATAAGCTTCGCATATGACAGAACCCCATCAACTGTGCATCTACACTAGAGGCTATAAAACTTCCGGATTTCGCTTTATCGGGCCATCGCTAAGTCTAGATTTTCTACTCATCAACATCATGGGAAAGTGGAATGCATTTTCTAGAGTGACAAAAAGGGACGGTAGTTCACTACCATT

left arm

right arm a

protospacer /PAM

*xylA_Eco_**

downstream (*cgl1890*)

upstream (*cgl1890*)

*kan*^R^ promoter

**(b)**


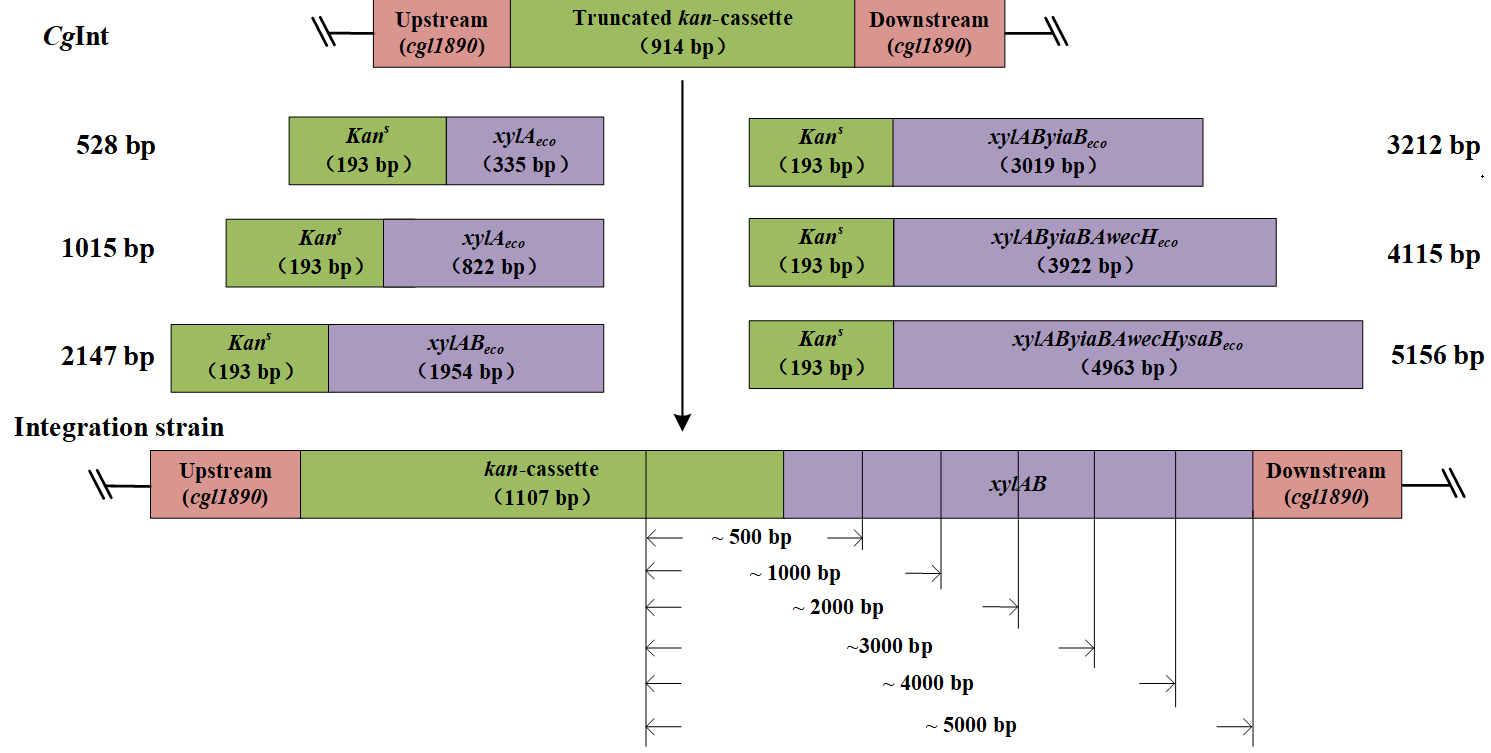


***Cg*Int:**

GACTAGTGGGGGTTTCTGCTGTTATGTTGCTGGCCATCGGGGTTGCATCTCCGGTGGCTCAAGCACAGGTGGAAGATCAATTTGAGCTTGTAGAAGAAATCACTGATGAGTAGTTTGCTGATGACGGTGTTGATTATGTTCCCAATAGGAATGCTCCAACAGTTGAGGAACAACTTGAGGATTACGAAACAGCACATCCAGAAGTAGTCGTTGAGTATCACGAGCAAGTCAACGATAGTAAAGATAATGTCGAGGAACTCCCGCTGCCTAAGCGGGACATAGTTGCAGGGGACATGCGTTCAGATGTTATCGAGTTACCGGAGGGGGTAAGCAAGGAGAAAGCTGACCAGCTAGAAGTTGCGGAAGCGCGACTTAACGAGGGTGCACGACTGATGGCAACCACCGGGTGTGAGGTTATGTGGCCAACGGGCTTCTCAGTTTGTGGCCGAATTCTTGACACCTATCGCCAGGAGGAAGCGGAACACGTAGAAAGCCAGTCCGCAGAAACGGTGCTGACCCCGGATGAATGTCAGCTACTGGGCTATCTGGACAAGGGAAAACGCAAGCGCAAAGAGAAAGCAGGTAGCTTGCAGTGGGCTTACATGGCGATAGCTAGACTGGGCGGTTTTATGGACAGCAAGCGAACCGGAATTGCCAGCTGGGGCGCCCTCTGGTAAGGTTGGGAAGCCCTGCAAAGTAAACTGGATGGCTTTCTTGCCGCCAAGGATCTGATGGCGCAGGGGATCAAGATCTGATCAAGAGACAGGATGAGGATCGTTTCGCATGATTGAACAAGATGGATTGCACGCAGGTTCTCCGGCCGCTTGGGTGGAGAGGCTATTCGGCTATGACTGGGCACAACAGACAATCGGCTGCTCTGATGCCGCCGTGTTCCGGCTGTCAGCGCAGGGGCGCCCGGTTCTTTTTGTCAAGACCGACCTGTCCGGTGCCCTGAATGAACTCCAAGACGAGGCAGCGCGGCTATCGTGGCTGGCCACGACGGGCGTTCCTTGCGCAGCTGTGCTCGACGTTGTCACTGAAGCGGGAAGGGACTGGCTGCTATTGGGCGAAGTGCCGGGGCAGGATCTCCTGTCATCTCACCTTGCTCCTGCCGAGAAAGTATCCATCATGGCTGATGCAATGCGGCGGCTGCATACGCTTGATCCGGCTACCTGCCCATTCGACCACCAAGCGAAACATCGCATCGAGCGAGCACGTACTCGGATGGAAGCCGGTCTTGTCGATCAGGATGATCTGGACGAAGAGCATCAGGGGCTCGCGCCAGCCGAACTGTTCGCCAGGCTCAAGGCGCGGATGCCCGACGGCGAGGATCTCGTCGTGACCCATGGCGATGCCTGCTTGCCGAATATCATGGTGGAAAATGGTGAGGCACTGGCGTTGGCGAAGCGTAAAAACTTCGCCAACCACGCTATGGCTGAGGCCGTAGTAGATAAAGCGGTGATTGATTATGGCTCATCGCCAGGAACCAGCTATTACAAGTTCGAGAAAACGGTGTACTTTCTAGATTGCAGAACTTATACATTCAATAAGAACTCAGGATGTAAAGAAATGCACGCTCCGCAATGGGTGACTATTATTTACAATCCTCATACTTTCACTGGAGCAAATTCGAACAGACCCAAGGGGGTAATTTCAGCATGGTGTAATTCAACCCCACCTGGTGGAATCGAACACGAGCCGGAAATTTCCCAATGTCCTGATCATGTGAATCTTTATAATAAGCTTCGCATATGACAGAACCCCATCAACTGTGCATCTACACTAGAGGCTATAAAACTTCCGGATTTCGCTTTATCGGGCCATCGCTAAGTCTAGATTTTCTACTCATCAACATCATGGGAAAGTGGAATGCATTTTCTAGAGTGACAAAAAGGGACGGTAGTTCACTACCATT

left arm

right arm a

protospacer /PAM

*kan*^R^ promoter

upstream (*cgl1890*)

**Integration strain:**

GAAGCGGAACACGTAGAAAGCCAGTCCGCAGAAACGGTGCTGACCCCGGATGAATGTCAGCTACTGGGCTATCTGGACAAGGGAAAACGCAAGCGCAAAGAGAAAGCAGGTAGCTTGCAGTGGGCTTACATGGCGATAGCTAGACTGGGCGGTTTTATGGACAGCAAGCGAACCGGAATTGCCAGCTGGGGCGCCCTCTGGTAAGGTTGGGAAGCCCTGCAAAGTAAACTGGATGGCTTTCTTGCCGCCAAGGATCTGATGGCGCAGGGGATCAAGATCTGATCAAGAGACAGGATGAGGATCGTTTCGCATGATTGAACAAGATGGATTGCACGCAGGTTCTCCGGCCGCTTGGGTGGAGAGGCTATTCGGCTATGACTGGGCACAACAGACAATCGGCTGCTCTGATGCCGCCGTGTTCCGGCTGTCAGCGCAGGGGCGCCCGGTTCTTTTTGTCAAGACCGACCTGTCCGGTGCCCTGAATGAACTCCAAGACGAGGCAGCGCGGCTATCGTGGCTGGCCACGACGGGCGTTCCTTGCGCAGCTGTGCTCGACGTTGTCACTGAAGCGGGAAGGGACTGGCTGCTATTGGGCGAAGTGCCGGGGCAGGATCTCCTGTCATCTCACCTTGCTCCTGCCGAGAAAGTATCCATCATGGCTGATGCAATGCGGCGGCTGCATACGCTTGATCCGGCTACCTGCCCATTCGACCACCAAGCGAAACATCGCATCGAGCGAGCACGTACTCGGATGGAAGCCGGTCTTGTCGATCAGGATGATCTGGACGAAGAGCATCAGGGGCTCGCGCCAGCCGAACTGTTCGCCAGGCTCAAGGCGCGGATGCCCGACGGCGAGGATCTCGTCGTGACCCATGGCGATGCCTGCTTGCCGAATATCATGGTGGAAAATGGCCGCTTTTCTGGATTCATCGACTGTGGCCGGCTGGGTGTGGCGGACCGCTATCAGGACATAGCGTTGGCTACCCGTGATATTGCTGAAGAGCTTGGCGGCGAATGGGCTGACCGCTTCCTCGTGCTTTACGGTATCGCCGCTCCCGATTCGCAGCGCATCGCCTTCTATCGCCTTCTTGACGAGTTCTTCTGAGCGGGACTCTGGGGTTCGCTAGAGGAT

*kan*^R^ promoter

*kan*^R^

CATGTGCCATTTTATTGCTTCCACGATGTGGATGTTTCCCCTGAGGGCGCGTCGTTAAAAGAGTACATCAATAATTTTGCGCAAATGGTTGATGTCCTGGCAGGCAAGCAAGAAGAGAGCGGCGTGAAGCTGCTGTGGGGAACGGCCAACTGCTTTACAAACCCTCGCTACGGCGCGGGTGCGGCGACGAACCCAGATCCTGAAGTCTTCAGCTGGGCGGCAACGCAAGTTGTTACAGCGATGGAAGCAACCCATAAATTGGGCGGTGAAAACTATGTCCTGTGGGGCGGTCGTGAAGGTTACGAAACGCTGTTAAATACCGACTTGCGTCAGGAGCGTGAACAACTGGGCCGCTTTATGCAGATGGTGGTTGAGCATAAACATAAAATCGGTTTCCAGGGCACGTTGCTTATCGAACCGAAACCGCAAGAACCGACCAAACATCAATATGATTACGATGCCGCGACGGTCTATGGCTTCCTGAAACAGTTTGGTCTGGAAAAAGAGATTAAACTGAACATTGAAGCTAACCACGCGACGCTGGCAGGTCACTCTTTCCATCATGAAATAGCCACCGCCATTGCGCTTGGCCTGTTCGGTTCTGTCGACGCCAACCGTGGCGATGCGCAACTGGGCTGGGACACCGACCAGTTCCCGAACAGTGTGGAAGAGAATGCGCTGGTGATGTATGAAATTCTCAAAGCAGGCGGTTTCACCACCGGTGGTCTGAACTTCGATGCCAAAGTACGTCGTCAAAGTACTGATAAATATGATCTGTTTTACGGTCATATCGGCGCGATGGATACGATGGCACTGGCGCTGAAAATTGCAGCGCGCATGATTGAAGATGGCGAGCTGGATAAACGCATCGCGCAGCGTTATTCCGGCTGGAATAGCGAATTGGGCCAGCAAATCCTGAAAGGCCAAATGTCACTGGCAGATTTAGCCAAATATGCTCAGGAACATCATTTGTCTCCGGTGCATCAGAGTGGTCGCCAGGAACAACTGGAAAATCTGGTAAACCATTATCTGTTCGACAAATAACGGCTAACTGTGCAGTCCGTTGGCCCGGTTATCGGTAGCGATACCGGGCATTTTTTTAAGGAACGATCGATATGTATATCGGGATAGATCTTGGCACCTCGGGCGTAAAAGTTATTTTGCTCAACGAGCAGGGTGAGGTGGTTGCTGCGCAAACGGAAAAGCTGACCGTTTCGCGCCCGCATCCACTCTGGTCGGAACAAGACCCGGAACAGTGGTGGCAGGCAACTGATCGCGCAATGAAAGCTCTGGGCGATCAGCATTCTCTGCAGGACGTTAAAGCATTGGGTATTGCCGGCCAGATGCACGGAGCAACCTTGCTGGATGCTCAGCAACGGGTGTTACGCCCTGCCATTTTGTGGAACGACGGGCGCTGTGCGCAAGAGTGCACTTTGCTGGAAGCGCGAGTTCCGCAATCGCGGGTGATTACCGGCAACCTGATGATGCCCGGATTTACTGCGCCTAAATTGCTATGGGTTCAGCGGCATGAGCCGGAGATATTCCGTCAAATCGACAAAGTATTATTACCGAAAGATTACTTGCGTCTGCGTATGACGGGGGAGTTTGCCAGCGATATGTCTGACGCAGCTGGCACCATGTGGCTGGATGTCGCAAAGCGTGACTGGAGTGACGTCATGCTGCAGGCTTGCGACTTATCTCGTGACCAGATGCCCGCATTATACGAAGGCAGCGAAATTACTGGTGCTTTGTTACCTGAAGTTGCGAAAGCGTGGGGTATGGCGACGGTGCCAGTTGTCGCAGGCGGTGGCGACAATGCAGCTGGTGCAGTTGGTGTGGGAATGGTTGATGCTAATCAGGCAATGTTATCGCTGGGGACGTCGGGGGTCTATTTTGCTGTCAGCGAAGGGTTCTTAAGCAAGCCAGAAAGCGCCGTACATAGCTTTTGCCATGCGCTACCGCAACGTTGGCATTTAATGTCTGTGATGCTGAGTGCAGCGTCGTGTCTGGATTGGGCCGCGAAATTAACCGGCCTGAGCAATGTCCCAGCTTTAATCGCTGCAGCTCAACAGGCTGATGAAAGTGCCGAGCCAGTTTGGTTTCTGCCTTATCTTTCCGGCGAGCGTACGCCACACAATAATCCCCAGGCGAAGGGGGTTTTCTTTGGTTTGACTCATCAACATGGCCCCAATGAACTGGCGCGAGCAGTGCTGGAAGGCGTGGGTTATGCGCTGGCAGATGGCATGGATGTCGTGCATGCCTGCGGTATTAAACCGCAAAGTGTTACGTTGATTGGGGGCGGGGCGCGTAGTGAGTACTGGCGTCAGATGCTGGCGGATATCAGCGGTCAGCAGCTCGATTACCGTACGGGGGGGGATGTGGGGCCAGCACTGGGCGCAGCAAGGCTGGCGCAGATCGCGGCGAATCCAGAGAAATCGCTCATTGAATTGTTGCCGCAACTACCGTTAGAACAGTCGCATCTACCAGATGCGCAGCGTTATGCCGCTTATCAGCCACGACGAGAAACGTTCCGTCGCCTCTATCAGCAACTTCTGCCATTAATGGCGTAAACGTTATCCCCTGCCTGACCGGGTGGGGGATAATTCACATCTATATATCTCAGTAATTAATTAATATTTAGTATGAATTTATTCTGAAAATCATTTGTTAATGGCATTTTTCAGTTTTGTCTTTCGTTGGTTACTCGTAATGTATCGCTGGTAGATATGGAGATCGTTATGAAAACCTCAAAGACTGTGGCAAAACTATTATTTGTTGTCGGGGCGCTGGTTTATCTGGTTGGGCTATGGATCTCATGCCCATTGTTAAGTGGAAAAGGCTATTTTCTTGGCGTGTTAATGACAGCAACTTTTGGCAACTATGCATATCTTCGCGCAGAAAAACTCGGGCAACTGGATGATTTTTTTACCCATATCTGCCAGTTAGTTGCGTTAATCACTATCGGTCTCTTGTTTATCGGTGTTTTAAACGCACCTATCAATACTTATGAAATGGTGATCTATCCCATCGCCTTTTTTGTCTGCTTGTTTGGTCAAATGCGTTTGTTTCGCTCGGCATGAGCAACATAAAGCTCTTACATATTCAGGAATGAAAGGAATACTGTGATGGACAACAAAATATCAACCTATTCACCGGCCTTTAGTATTGTGTCATGGATAGCTCTCGTTGGTGGTATCGTTACCTATCTGTTAGGGCTATGGAATGCAGAGATGCAGTTAAATGAAAAAGGATATTATTTTGCCGTACTGGTATTAGGACTGTTTTCTGCGGCGTCTTATCAAAAGACCGTTCGGGACAAGTATGAAGGCATACCGACCACTTCCATTTATTATATGACCTGCCTGACTGTCTTTATTATCTCTGTTGCATTACTGATGGTAGGTCTGTGGAATGCGACATTATTACTCAGCGAAAAAGGTTTTTATGGACTGGCTTTCTTCTTAAGCTTGTTTGGTGCAGTAGCGGTGCAGAAAAATATTCGTGATGCCGGAATAAACCCACCAAAAGAAACACAGGTTACCCAGGAAGAATACAGCGAATAACTCACGTAAGCCCGGTCAGTCCAATGTGACCGGGCTTTTACTTAACTCACTAATCTGTTTCTGTCGATTCGTTGTACCAGCATAGAAAGTAACAAACTCGCTGCCAACGTCGCGCAAAAGATCCAAATAATATCCAGTATTGGCCAATTTTTAAGCTCAATTCCCCGGGTGCGCAGCGCATGGATAATCAAGGCGTGGAATCCGTATATACCCAATGAATGGCGGGAGATTAAGCCAAGTCCGCGAATGGTACGCGTATCCAGCGTGTTTTTAACCAGAGTCAATAGCGCGATTGCGCAGATAAAAACCATCGGCCCACAGTAAAGATACCAGGTATCGGCAAAATTTCCGCGCCACTGCAATTCATATAATGTCCCGCGAGAGATAATAAAAACCCCCGTCGCAAACAGCGCGGCGCTCACCCACGACAGTGCTTTATGCTGTGTGTCCATCATCCCTATAGCGCGGCCCAACATGCCATACAGAATGTAGTAAAAAGTATCGCCATTGATATATAAGTTAATTGGCAGCCATTCAAAACCGTCAATTTTCTGCGGCACTGTGTTTGGGTTAGCGATAATGCCAATCACCGCCATTAGCACCAGCAACATTTTTCCGCCGACGTTCTTCACCTGAATCAGCGGTGAAACCAGATAAATCACCGCAATCGCGAAGAAAAACCACAAGTGGTAAAACACTGGCTTTTGCAGCAGGTTTTTCAGCGCTAACTCCATATTGATGGAGGTAAACAGCGCAATGTAGAGCAGTGCGATTGCGCTATAAAAAATCAGACATAAGCCGATACGCAAGAAATGGCGCGGCTGGGCGCTGCGTTCGCCAAAAAAGAGATAGCCGGAAATCATGAAAAATAGCGGCACGCTGACACGAGAGGCAGAATTCAGAACATTGGCGATATCCCATGTGACGGGGCTAACACTATGAGCATTGGTCACATACCAGGTAGTGGTGTGAATCATCACCACCATTAAACACGCTATCCCTCGCAGGTTATCAATCCAGTAAATTTTGGGCTGCATCTGTGTCTCTGTATCTGGTTAAAAAAAGTCTGACCGATAAATCATTTGGTTGGCGCACTGGAATAATCTGAGTTTTATCACTACAGCTTATAGAGGCTTAAGGAAATTCGTAAGATATCAGCCACTATACCGATATAAATAATAAGACTCACCTGCAAACCAGACGGTAATTTAATGATGATGAACGCTTTCTTTCCGGCAATGGCGCTTATGGTGCTAGTGGGTTGTTCTATACCGTCACCCGTGCAGAAAGCACAACGGGTAAAGGTTGATCCTCTGCGTTCGTTGAATATGGAAGCGTTATGCAAGGATCAGGCGGCAAAACGTTATAACACC

UH (*kan*^R^)

terminal of stepwisely extended sequences integrated with supplementary *kan*^R^ sequence

AACTTCGCCAACCACGCTATGGCTGAGGCCGTAGTAGATAAAGCGGTGATTGATTATGGCTCATCGCCAGGAACCAGCTATTACAAGTTCGAGAAAACGGTGTACTTTCTAGATTGCAGAACTTATACATTCAATAAGAACTCAGGATGTAAAGAAATGCACGCTCCGCAATGGGTGACTATTATTTACAATCCTCATACTTTCACTGGAGCAAATTCGAACAGACCCAAGGGGGTAATTTCAGCATGGTGTAATTCAACCCCACCTGGTGGAATCGAACACGAGCCGGAAATTTCCCAATGTCCTGATCATGTGAATCTTTATAATAAGCTTCGCATATGACAGAACCCCATCAACTGTGCATCTACACTAGAGGCTATAAAACTTCCGGATTTCGCTTTATCGGGCCATCGCTAAGTCTAGATTTTCTACTCATCAACATCATGGGAAAGTGGAATGCATTTTCTAGAGTGACAAAAAGGGACGGTAGTTCACTACCATT

DH (*cgl1890*)

**Fig. S1 Diagrams of model strains *Cg*Del and *Cg*Int and the corresponding deletion strain and integration strain, and their annotated sequences. a**: *Cg*Del, deletion strain and relevant sequences. **b**: *Cg*Int, integration strains and relevant sequences. Extended sequences for integration are genes *xylA*, *xylB*, *yiaB*, *yiaA*, *wecH*, and *ysaB* from *E. coli*.

**
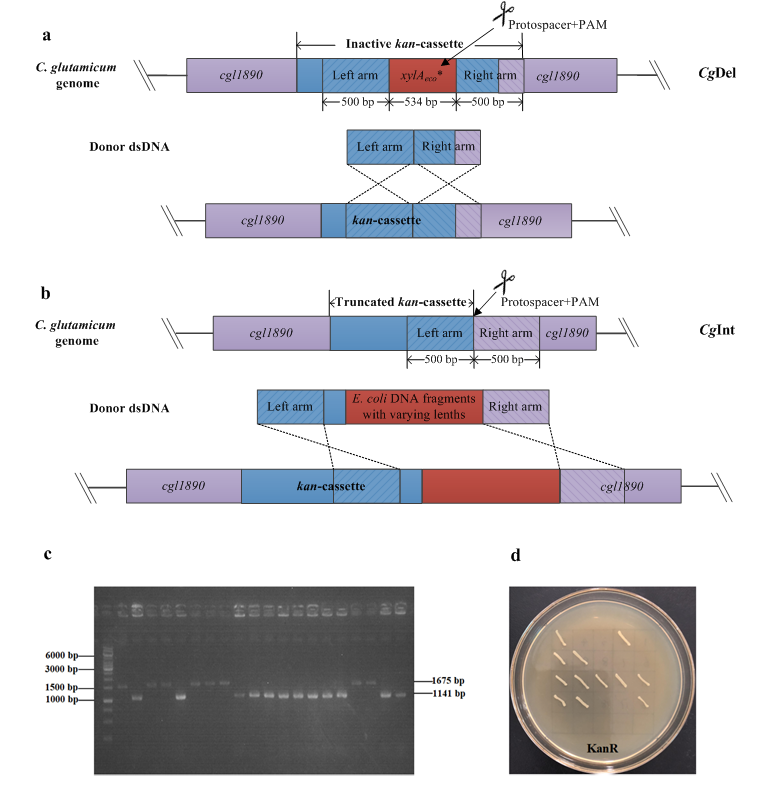
**

**Fig. S2** **Schematic illustrations of the rapid identification of gene deletion and integration for the model strain *Cg*Del and *Cg*Int, respectively. a**: *Cg*Del, i.e., a 534 bp partial sequence of the *E. coli* *xylA* gene (*xylA_Eco_**) (Fig. S1a) is deleted, this strain confers kanamycin resistance. **b**: *Cg*Int, i.e., the 193 bp C-terminal of the Kan^R^ sequence is complemented with different lengths of extra sequences (Fig. S1b); this strain confers kanamycin resistance. **c**: Colony PCR verification of *xylA_Eco_**-deleted strains. **d**: Growth of corresponding colonies inoculated on an agar plate supplemented with kanamycin, indicating the feasibility of kanamycin selection for genome-edited strains.


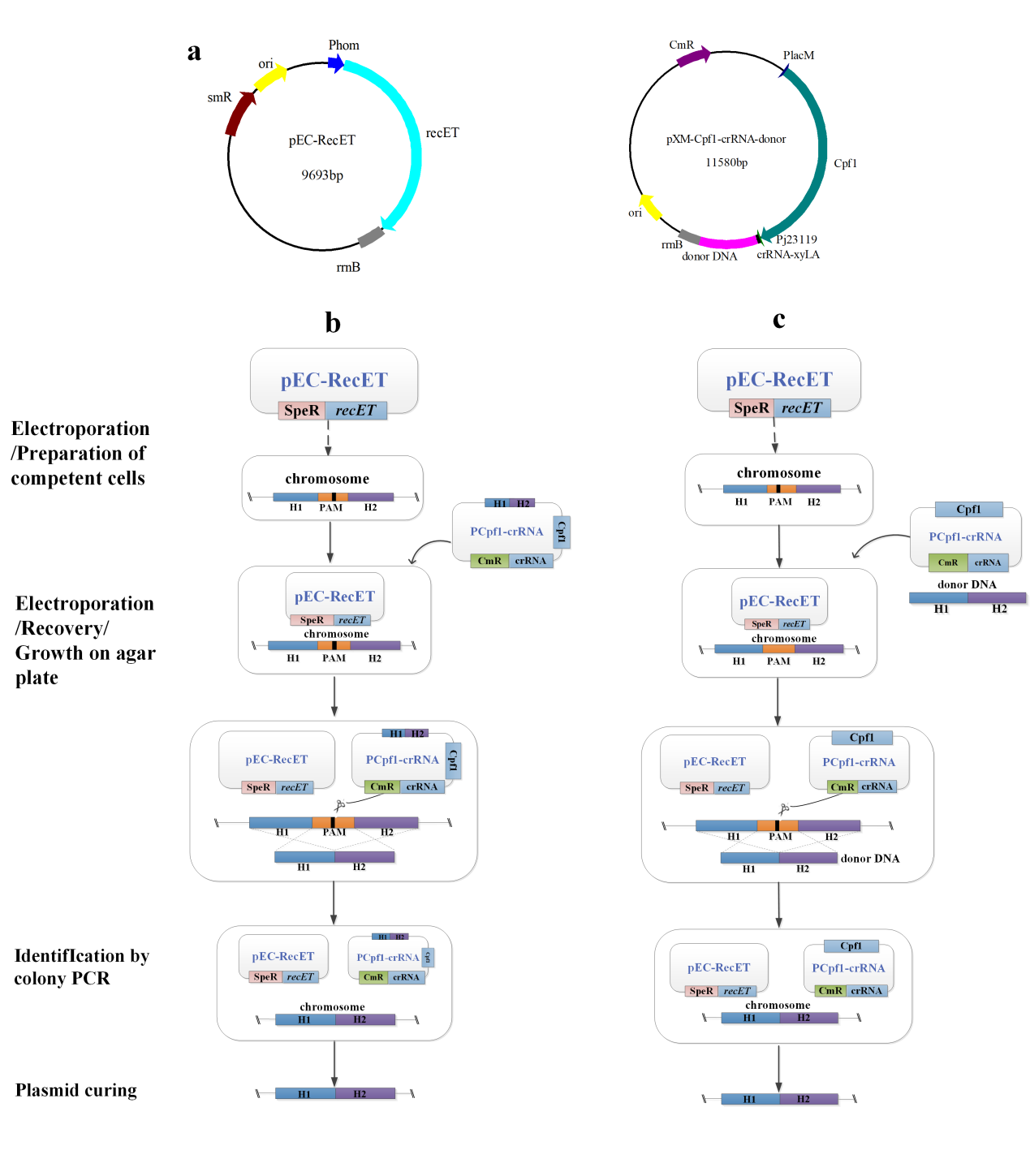


**Fig. S3** **Diagram of one-step RecET-assisted CRISPR-Cpf1 genome editing system. a**: Plasmid map of pEC-RecET and pXM-Cpf1-crRNA-donor. **b**: Workflow of one-step genome editing using a plasmid-borne DNA template. **c**: Workflow of one-step genome editing using linear DNA template.


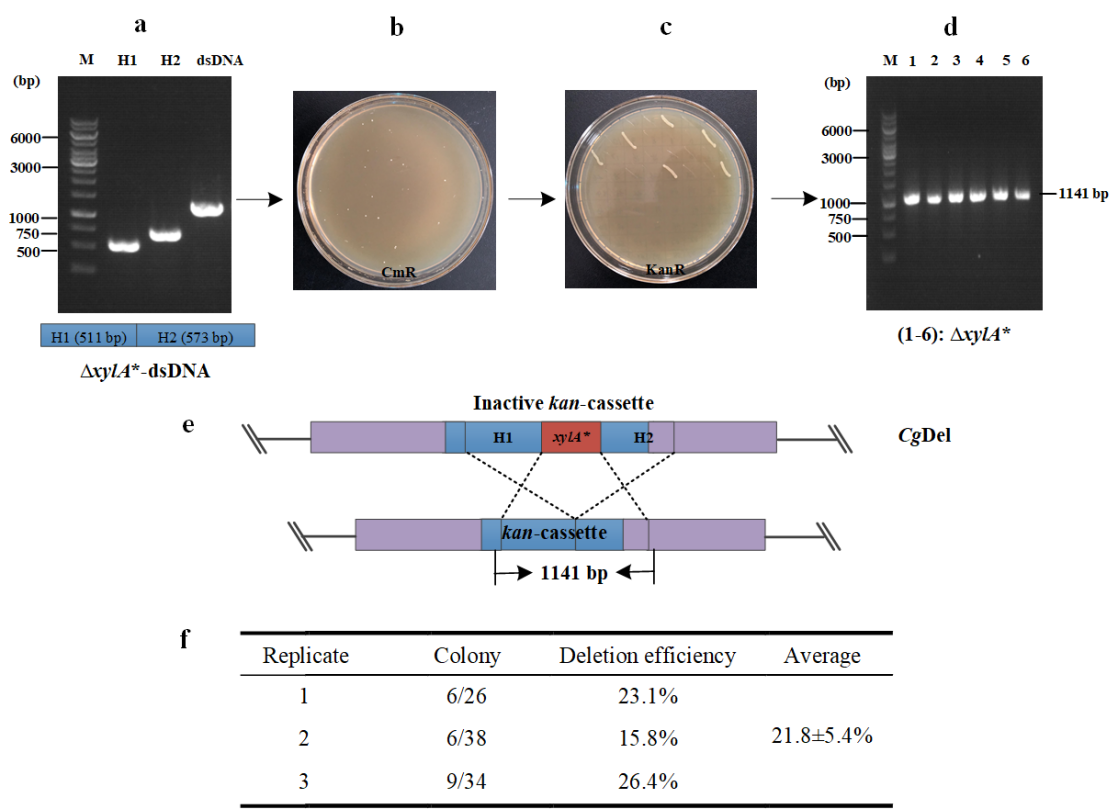


**Fig. S4 Gene deletion by the one-step system using linear DNA as a template. a**: Linear fragment construction for deleting the *xylA_Eco_** from *Cg*Del. **b**: Transformants present on an agar plate. **c**: Edited colonies grew on a kanamycin-supplemented agar plate. **d**: Colony PCR identification. **e**: Sketch of gene deletion conferring kanamycin resistance. **f**: Gene deletion efficiency.


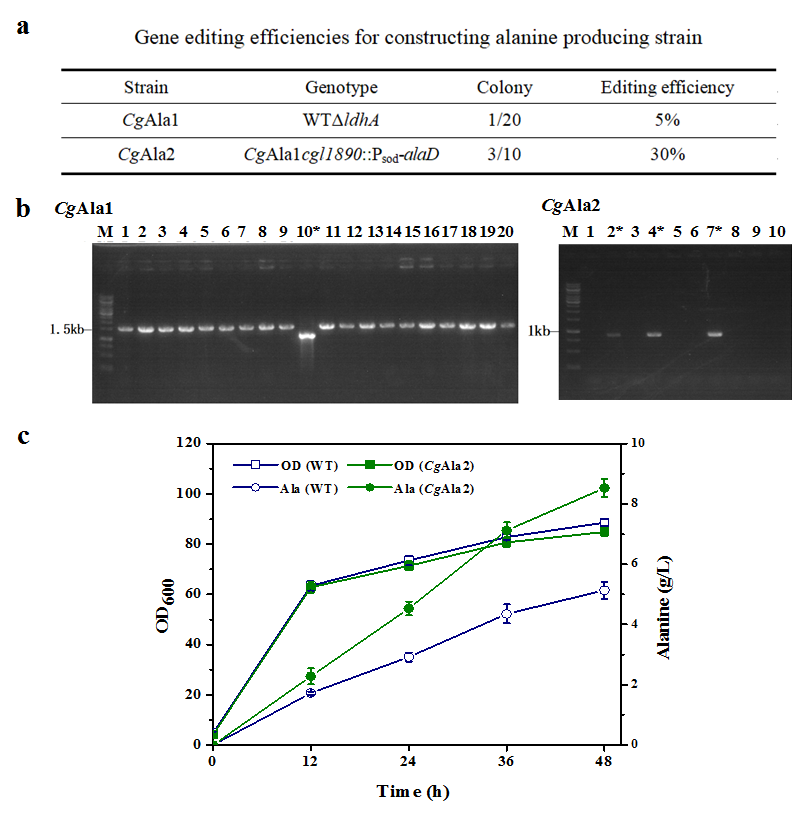


**Fig. S5** **Application of one-step CRISPR-Cpf1 system for the construction of the alanine producing strain. a**: Genomic manipulations and the corresponding editing efficiencies. **b**: Colony PCR identification of *ldhA* deletion (obtaining strain *Cg*Ala1) and subsequent *alaD_Bsu_* integration (obtaining strain *Cg*Ala2). **c**: Results of the shake-flask fermentation of the two strains, the optical density OD_600_ and alanine production are presented. Experiments were performed in triplicates. Values are presented as mean ± SD.

**
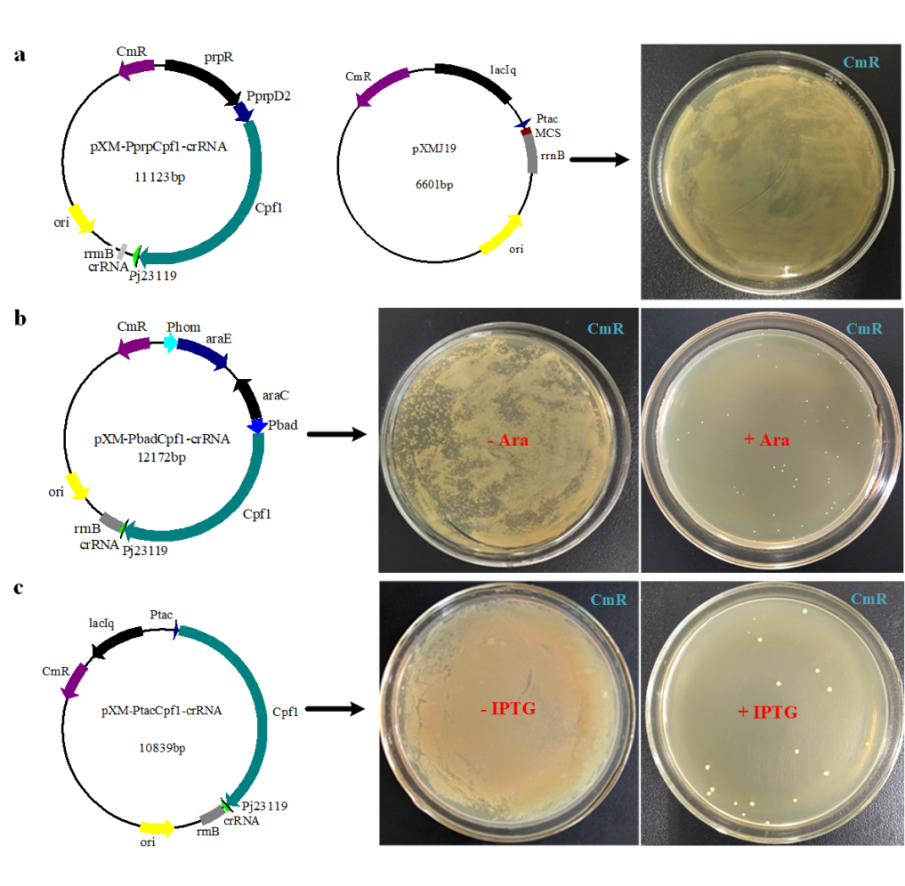
**

**Fig. S6** **Different inducible systems for *Fn*Cpf1 expression. a**: Plasmid map of the propionate-inducible expression system and the transformation of pXMJ19 control plasmid. **b**: Plasmid map and transformation results of the arabinose-inducible system. **c**: Plasmid map and transformation results of the IPTG-inducible system.

**
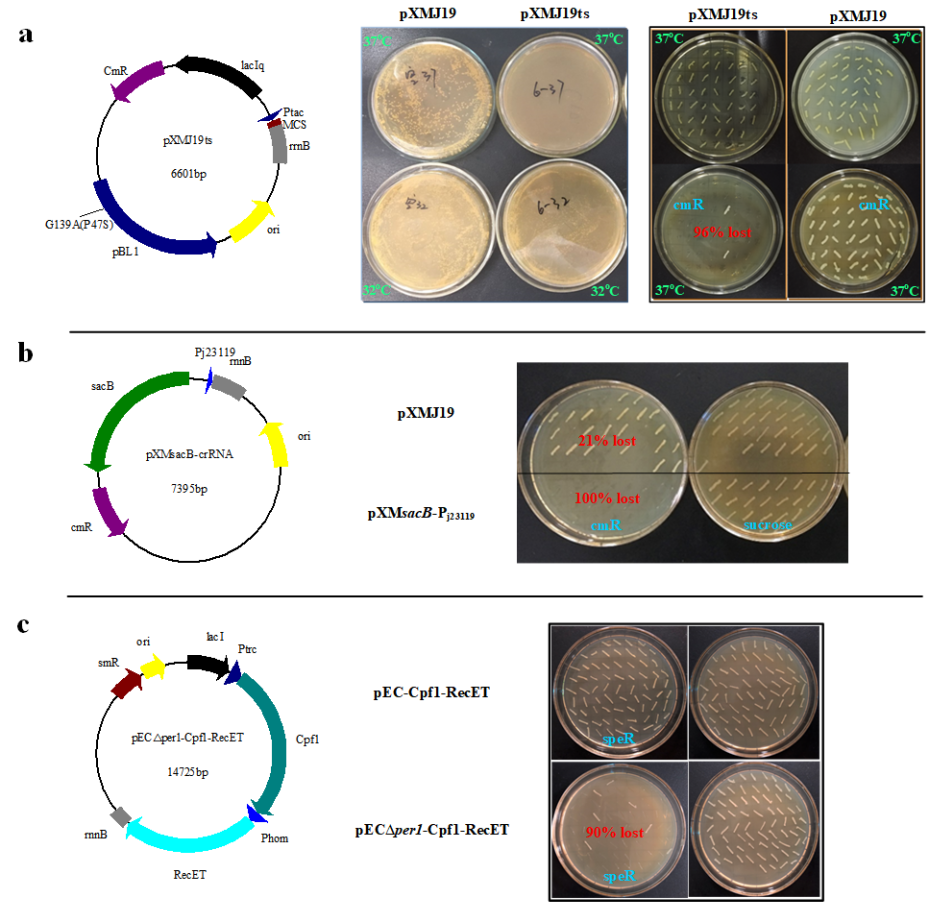
**

**Fig. S7** **Strategies for curing pXMJ19 and pEC-XK99E derived plasmids. a**: Curing of pXMJ19ts by the temperature elevation. The transformation efficiency of pXMJ19ts was markedly reduced, even at 32 ^o^C, compared to that of pXMJ19. The curing of pXMJ19ts reached 96% after one culture in a chloramphenicol-free medium, while pXMJ19 could not be cured under the same condition. **b**: Curing of *sacB*-harboring plasmid by sucrose addition. The curing of pXM*sacB*-P_j23119_ and pXMJ19 reached 100% and 21%, respectively, after one culture supplemented with 1.5% (w/v) sucrose. **c**: Curing of plasmid pECΔ*per1*-Cpf1-RecET. The curing of pECΔ*per1*-Cpf1-RecET reached 90% after one culture in the absence of spectinomycin, while pEC-Cpf1-RecET could not be cured under the same condition.

**
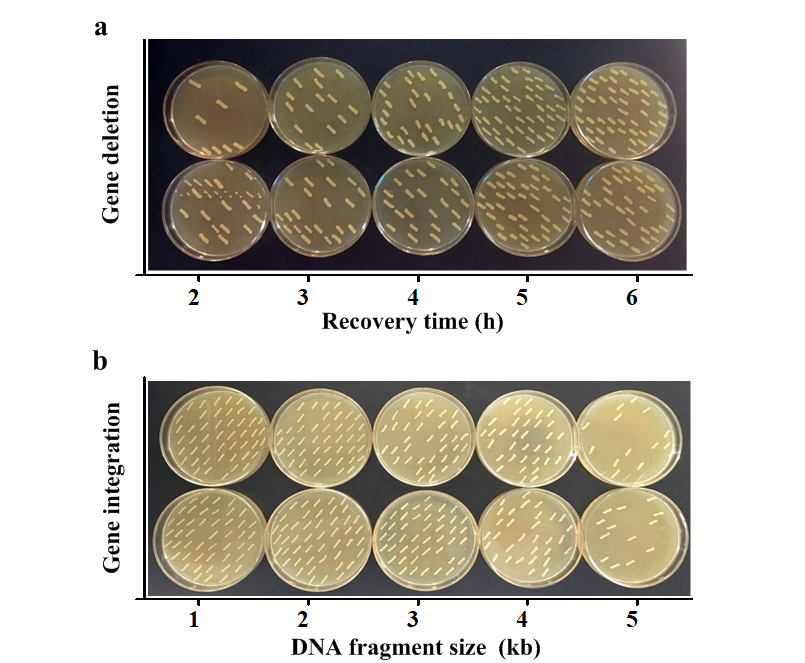
**

**Fig. S8** **The growth of genome-edited cells by RAPID, identified by kanamycin selection on agar plates. a**: The deletion of 534 bp *xylA_Eco_** from the *Cg*Del genome under varying recovery time (2-6 h). **b**: The integration of DNA fragments with lengths of 1–5 kb, into the *Cg*Int genome.


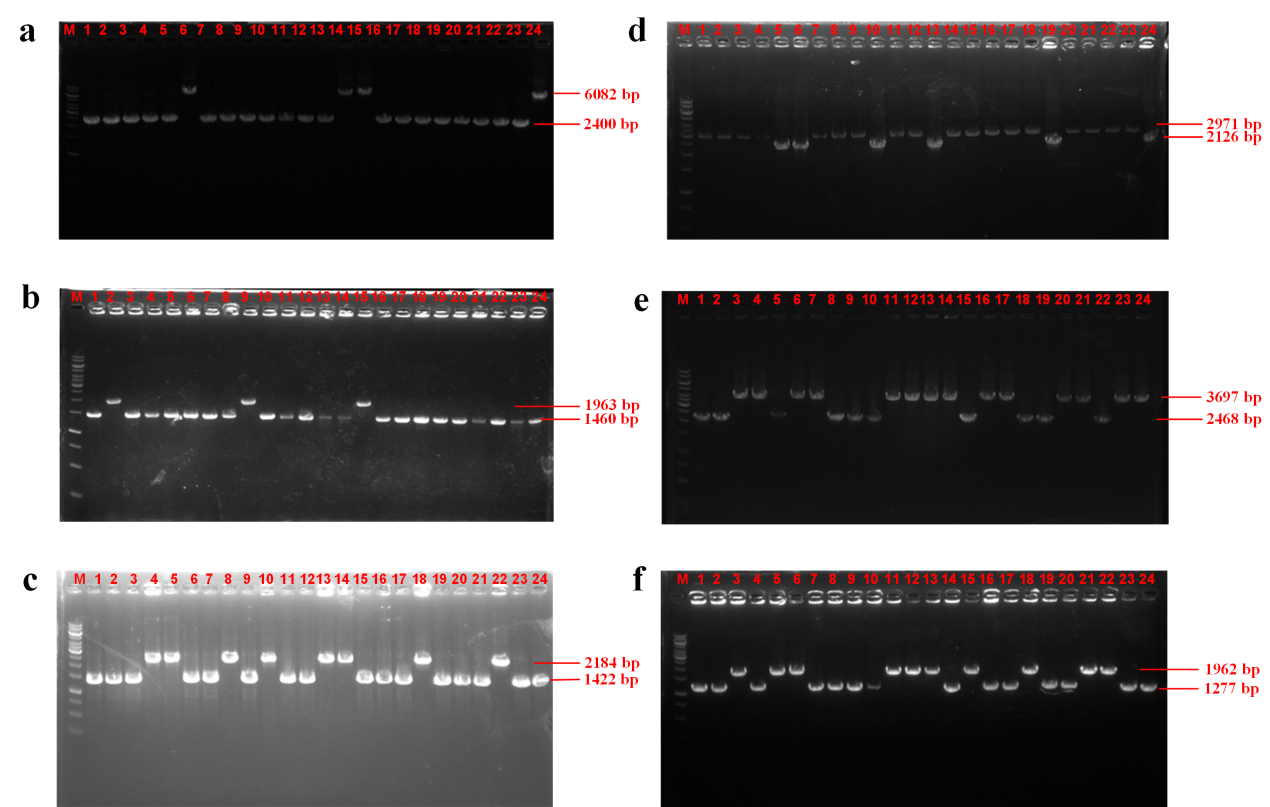


**Fig. S9 Colony PCR identification of gene deletion and integration using RAPID for construction of D-PA producers. a**: *ilvBNC* insertion; **b**: *ilvE* deletion; **c**: *avtA* deletion; **d**: *aspB* insertion; **e**: *aspA_Eco_* insertion; and **f**: *ilvA* deletion.
